# Indole Alkaloids Inhibiting Neural Stem Cell from *Uncaria rhynchophylla*

**DOI:** 10.1007/s13659-017-0141-y

**Published:** 2017-09-26

**Authors:** Xin Wei, Li-Ping Jiang, Ying Guo, Afsar Khan, Ya-Ping Liu, Hao-Fei Yu, Bei Wang, Cai-Feng Ding, Pei-Feng Zhu, Ying-Ying Chen, Yun-Li Zhao, Yong-Bing Chen, Yi-Fen Wang, Xiao-Dong Luo

**Affiliations:** 1 State Key Laboratory of Phytochemistry and Plant Resources in West China, Kunming Institute of Botany, Chinese Academy of Sciences, Kunming, 650201 People’s Republic of China; 2 Key Laboratory of Animal Models and Human Disease Mechanisms, Kunming Institute of Zoology, Chinese Academy of Sciences, Kunming, 650223 People’s Republic of China; 3 Department of Chemistry, COMSATS Institute of Information Technology, Abbottabad, 22060 Pakistan; 4 University of Chinese Academy of Sciences, Beijing, 100049 People’s Republic of China; 5 Yunnan Key Laboratory of Natural Medicinal Chemistry, Kunming, 650201 People’s Republic of China

**Keywords:** *Uncaria rhynchophylla*, Indole alkaloids, NSCs proliferation

## Abstract

*Uncaria rhynchophylla* is commonly recognized as a traditional treatment for dizziness, cerebrovascular diseases, and nervous disorders in China. Previously, the neuro-protective activities of the alkaloids from *U. rhynchophylla* were intensively reported. In current work, three new indole alkaloids (**1–3**), identified as geissoschizic acid (**1**), geissoschizic acid *N*_4_-oxide (**2**), and 3*β*-sitsirikine *N*_4_-oxide (**3**), as well as 26 known analogues were isolated from *U. rhynchophylla*. However, in the neural stem cells (NSCs) proliferation assay for all isolated compounds, geissoschizic acid (**1**), geissoschizic acid *N*_4_-oxide (**2**), isocorynoxeine (**6**), isorhynchophylline (**7**), (4*S*)-akuammigine *N*-oxide (**8**), and (4*S*)-rhynchophylline *N*-oxide (**10**) showed unexpected inhibitory activities at 10 μM. Unlike previous neuro-protective reports, as a warning or caution, our finding showcased a clue for possible NSCs toxicity and the neural lesions risk of *U. rhynchophylla*, while the structure–activity relationships of the isolated compounds were discussed also.

**Electronic supplementary material:**

The online version of this article (doi:10.1007/s13659-017-0141-y) contains supplementary material, which is available to authorized users.

## Introduction

The dried stem and hook of *Uncaria rhynchophylla*, named as Gou-teng or Cat’s claw, was used for treatment of dizziness, cerebrovascular diseases, and nervous disorders [1, 2]. Up to now, detailed phytochemical research resulted in more than 90 indole alkaloids from *U. rhynchophylla* [2]. Its alkaloids, commonly recognized as bioactive ingredients, are responsible for the pharmacological activities closely related to neuro-protective effects [2]. Previously, the neuro-protective activities of the total alkaloids and the main monomeric indoles from *U. rhynchophylla* were reported [3–7]. Over the past few years, continuing research on neuro-protective activities of *U. rhynchophylla* have culminated in considerable discoveries [8–11].

In adult nervous system, neural stem cells (NSCs) can self-renew and differentiate into almost all types of neural cells [12, 13]. Thus, countless new neurons are sustained throughout adulthood [12]. Recent medical research suggested many neurodegenerative diseases, such as Parkinson’s disease or Alzheimer’s disease (AD), may benefit from NSCs transplantation as well as its differentiation and proliferation capacity [14]. Enlightened by the importance of NSCs, small molecules and natural products promoting the NSCs differentiation and proliferation been intensively investigated [15, 16]. However, a very little work been focused on the substances or compounds with inhibitory effects, which may contribute to the possible risk of neural lesions [17].

**Fig. 1:**
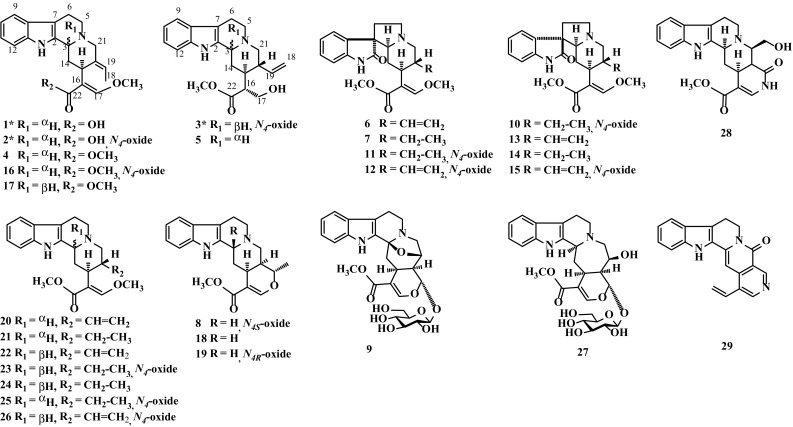
Structures of compounds **1**–**29**

As our ongoing search for novel and bioactive alkaloids as well as further NSCs related evaluation [16, 18], the detailed chemical investigation on hook-bearing branches of *U. rhynchophylla* was carried out. As a result, three new indole alkaloids, geissoschizic acid (**1**), geissoschizic acid *N*_4_-oxide (**2**), 3*β*-sitsirikine *N*_4_-oxide (**3**), along with 26 known analogues (Fig. 1), geissoschizine methyl ether (**4**) [19], sitsirikine (**5**) [20], isocorynoxeine (**6**) [21], isorhynchophylline (**7**) [21], (4S)-akuammigine *N*-oxide (**8**) [22], cadambine (**9**) [23], (4*S*)-rhynchophylline *N*-oxide (**10**) [24], isorhynchophylline *N*_4_-oxide (**11**) [25], (4*S*)-isocorynoxeine *N*-oxide (**12**) [24], corynoxeine (**13**) [21], rhynchophylline (**14**) [21], (4*S*)-corynoxeine *N*-oxide (**15**) [24], geissoschizine methyl ether *N*_4_-oxide (**16**) [26], 3-*epi*-geissoschizine methyl ether (**17**) [21], akuammigine (**18**) [27], (4*R*)-akuammigine *N*-oxide (**19**) [22], corynantheine (**20**) [28], dihydrocorynantheine (**21**) [28], hirsuteine (**22**) [23], hirsutine *N*-oxide (**23**) [29], hirsutine (**24**) [23], dihydrocorynantheine *N*-oxide (**25**) [30], hirsuteine *N*-oxide (**26**) [29], 3*α*-dihydrocadambine (**27**) [31], nitrocadambine B (**28**) [32], and augustine (**29**) [33], were isolated. NSCs proliferation assay for all the compounds (**1**–**29**) with DMSO and puromycin as the control groups exhibited the unexpected inhibitory activities of compounds **1–2**, **6**–**8**, and **10** at 10 μM (Table 1). Besides reported neuro-protective activities, the tested results shed a light on the possible NSCs toxicity and and the neural lesions risk of *U. rhynchophylla*, as a warning or caution. Meanwhile, the structure–activity relationships of the compounds mentioned above were discussed herein by structural analysis.

**Table 1:** NSCs Proliferation of Compounds **1**–**2**, **6**–**8**, and **10** at 10 μM

Compound	Proliferation (%)	Compound	Proliferation (%)
**1**	56.7	**8**	56.5
**2**	87.8	**10**	76.5
**6**	73.2	Puromycin^a^	17.1
**7**	68.8	DMSO^b^	100.0

^a^Positive control (10 μM)

^b^Negative control

## Results and Discussion

**Fig. 2:**
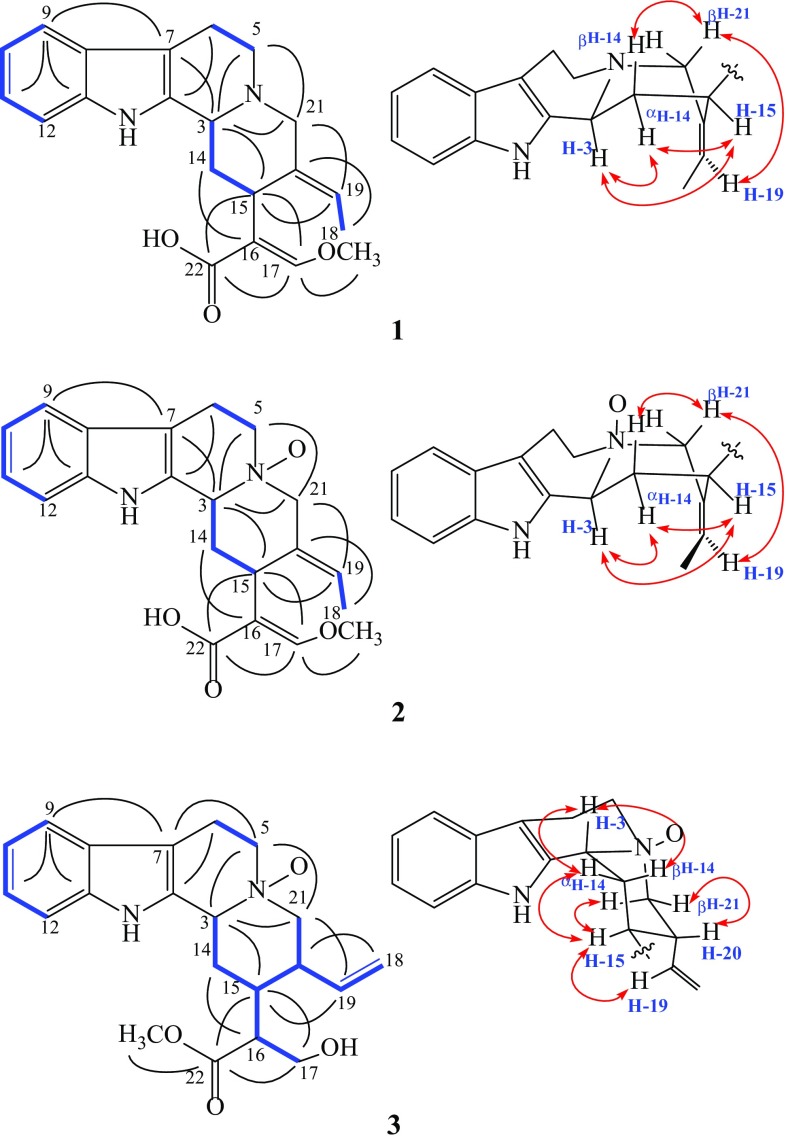
Selective HMBC (→), ^1^H-^1^H COSY (), and ROESY ( ) correlations of compounds **1**–**3**

**Fig. 3:**
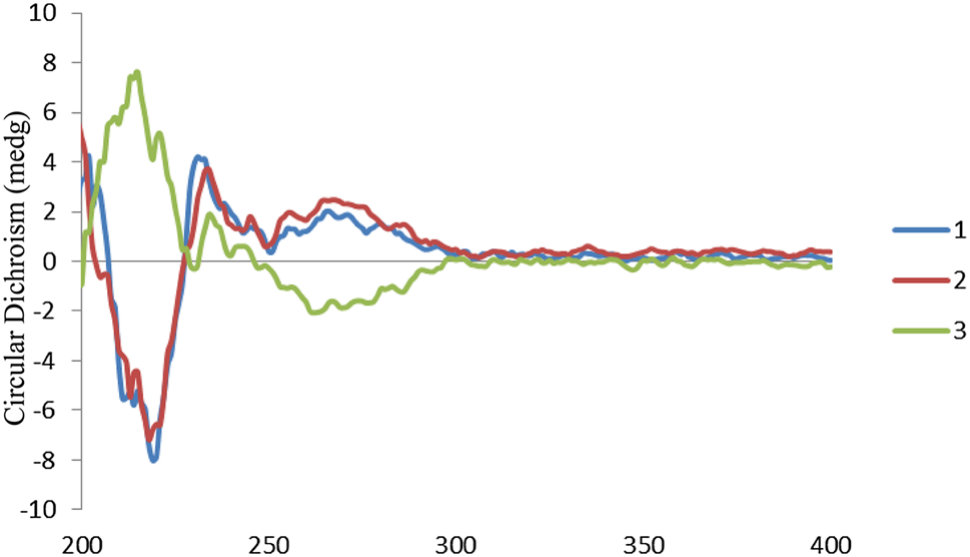
ECD spectra of compounds **1–3**

Compound **1** was assigned a molecular formula C_21_H_24_N_2_O_3_ from HRESIMS ion at *m/z* 353.1852 [M+H]^+^ (calcd for C_21_H_25_N_2_O_3_, 353.1860). The UV spectrum showed absorption maxima characteristic of an indolenine chromophore (269, 223, 207 nm) [34]. The IR spectrum showed absorption bands at 3421 (N–H) and 1644 cm^−1^ (C=O). The ^1^H NMR spectroscopic data (Table 2) revealed signals for non-substituted a ring of indole system [34], a methoxyl group, an olefinic proton, and a methyl group. The ^13^C NMR and DEPT spectra showed a total of 21 carbon resonances, including one methyl, one methoxyl, four methylenes, eight methines, and seven quaternary carbons. The ^1^H and ^13^C NMR spectroscopic data of **1** (Table 2) was similar to those of geissoschizine methyl ether (**4**) [19] except methoxyl group in **4**, substituent by -OH in **1**, consistent with its molecular formula. The typical chemical shift of -OCH_3_ (*δ*_C_ 61.4) at C-17, was present in ^13^C NMR spectrum of **1**, and further supported by correlation of *δ*_H_ 3.74 (s, -OCH_3_) with *δ*_C_ 158.5 (C-17) in its HMBC spectrum (Fig. 2), which suggested that **1** was hydrolysate of **4**. In the tetracyclic indole alkaloid (**1**), the absolute configuration of C-3 was determined as *S* according to a positive cotton effect at 267 nm, while negative Cotton effect for *R* (Fig. 3) [35]. Moreover, the NOE correlation of *δ*_H_ 4.20 (H-3) with *δ*_H_ 4.09 (H-15) in its ROESY spectrum, established C-15*S* (Fig. 2). The double bond of C-19/20 was *E* by the ROESY correlations of *δ*_H_ 4.07 (H-21)/5.53 (H-19) (Fig. 2). Meanwhile, the *trans* double bond of C-16/17 was indicated by the upfield chemical shift of olefinic proton at *δ*_H_ 7.20 (s, H-17), while that of the *cis* compounds at *δ*_H_ 7.89 (s, H-17) [26, 36].

**Table 2:** ^1^H and ^13^C NMR Spectroscopic Data of **1**–**3** (*δ* in ppm, *J* in Hz)

Position	**1**		**2**		**3**	
	*δ*_H_^a^	*δ*_C_^c^	*δ*_H_^a^	*δ*_C_^d^	*δ*_H_^b^	*δ*_C_^d^
**2**		131.9		131.0		130.3
**3**	4.20 (dd, 3.7, 11.7)	57.7	4.29 (d, 12.2)	75.0	4.59 (br. s)	71.6
**5**	3.34 (dd, 5.1, 12.0)	51.1	3.76 (overlap)	63.2	3.66 (overlap)	69.4
	3.16 (ddd, 5.1, 7.4, 12.0)		3.56 (m)		3.66 (overlap)	
**6**	2.99 (m)	20.6	3.29 (overlap)	18.9	3.09 (m)	20.6
	2.88 (dt, 5.1, 5.1, 10.9)		2.88 (br. d, 14.1)		3.02 (m)	
**7**		107.5		106.9		106.8
**8**		127.5		127.9		127.6
**9**	7.41 (d, 7.8)	119.0	7.46 (d, 7.8)	119.6	7.36 (d, 7.8)	119.1
**10**	7.00 (t, 7.8)	120.3	7.00 (t, 7.8)	120.7	6.96 (t, 7.8)	120.7
**11**	7.09 (t, 7.8)	123.0	7.08 (t, 7.8)	123.3	7.05 (t, 7.8)	123.4
**12**	7.30 (d, 7.8)	112.2	7.28 (d, 7.8)	112.5	7.28 (d, 7.8)	112.6
**13**		138.5		138.9		138.9
**H**_**α**_ **-14**	2.42 (ddd, 3.7, 7.9, 14.0)	32.6	2.23 (m)	34.6	2.26 (ddd, 2.2, 12.8, 14.2)	25.1
**H**_**β**_ **-14**	2.30 (ddd, 8.9, 11.7, 14.0)		2.34 (m)		2.60 (dt, 5.0, 14.2)	
**15**	4.09 (d, 7.9)	33.6	4.10 (d, 6.9)	36.2	1.47 (tt, 1.5, 12.2, 12.8)	35.1
**16**		117.5		117.7	2.78 (ddd,2.8, 6.1, 8.5)	49.1
**17**	7.20 (s)	158.5	7.16 (s)	158.1	3.92 (dd, 8.4, 11.0)	62.3
					3.52 (dd, 6.3, 11.0)	
**18**	1.64 (d, 6.6)	13.5	1.68 (d, 6.4)	14.3	5.18 (dd, 1.8, 17.1)	120.4
					5.08 (dd, 1.8, 10.0)	
**19**	5.53 (q, 6.6)	126.5	5.73 (q, 6.4)	131.4	5.31 (m)	137.4
**20**		133.0		132.8	3.04 (m)	40.7
**H**_**α**_ **-21**	3.62 (d, 13.1)	61.1	5.07 (d, 11.7)	78.7	3.32 (t, 11.9)	63.0
**H**_**β**_ **-21**	4.07 (d, 13.1)		3.76 (overlap)		2.90 (dd, 3.5, 11.9)	
**22**		174.7		175.5		174.4
**OMe**_**-**_ **17**	3.74 (s)	61.4	3.72 (s)	61.6		
**OMe**_**-**_ **22**					3.64 (s)	52.0

^a^Recorded at 400 MHz in CD_3_OD

^b^Recorded at 600 MHz in CD_3_OD

^c^Recorded at 100 MHz in CD_3_OD

^d^Recorded at 150 MHz in CD_3_OD

The molecular formula of **2** was assigned as C_21_H_24_N_2_O_4_ on the basis of its HRESIMS ion at *m/z* 369.1806 [M+H]^+^ (calcd for C_21_H_25_N_2_O_4_, 369.1809), 16 mass units more than **1**. Comparison the ^1^H and ^13^C NMR spectroscopic data of **1** with those of **2** (Table 2) showed both the compounds contain similar structural features except the downfield shifts of C-3 (*δ*_C_ 75.0, +17.3 ppm), C-5 (*δ*_C_ 63.2, +12.1 ppm), and C-21 (*δ*_C_ 78.7, +17.6 ppm) in **2**, which suggested **2** was an *N*_4_-oxide derivative of **1 [** 26]. Moreover, compound **2** shared the same configurations with **1** from their same ECD spectral curves (Fig. 3) and positive signs of their optical rotation, which also supported by detailed NOE correlations (Fig. 2) in the ROESY spectrum of **2**.

3*β*-sitsirikine *N*_4_-oxide (**3)** was established a molecular formula C_21_H_26_N_2_O_4_ on the basis of HRESIMS ion at *m/z* 371.1960 [M+H]^+^ (calcd for C_21_H_27_N_2_O_4_, 371.1965) and the comprehensive analysis of ^13^C NMR data (Table 2), indicating 10 degrees of unsaturation. Its ^1^H NMR and ^13^CNMR spectroscopic data (Table 2) were similar to sitsirikine (**5**) [20]. The visible differences were the downfield shifts of C-3 (*δ*_C_ 71.6, +10.2 ppm), C-5 (*δ*_C_ 69.4, +7.2 ppm), and C-21 (*δ*_C_ 63.0, +9.0 ppm) in **3** as well as 16 mass units more than **5**, which suggested that **3** was an *N*_4_-oxide derivative of sitsirikine (**5**) [20, 26]. Contrary to **1** and **2**, a negative Cotton effect at 262 nm revealed the *R* configuration of C-3 (Fig. 3) [35]. In its ROESY spectrum, NOE correlations of H-3 with both C-14 parahydrogens further supported that the H-3 is in a procumbent equatorial bond (*β*-orientation) (Fig. 2) [29]. Besides, the NOE correlation of *δ*_H_ 1.47 (H-15) with *δ*_H_ 5.31 (H-19) indicated H-15 and H-19 at the same side (*α*-orientation) while the H-20 at another side, owing to the *α*-orientation of H-15 from its biosynthetic consideration [2]. The C-16*R* of natural sitsirikine (**5**) have been established on the basis of coupling constants data and chemical methods [20, 37]. The H-17a and H-17b appeared as doublet of doublets at *δ*_H_ 3.97 (*J* = 11.0, 8.0 Hz) and 3.76 (*J* = 11.0, 6.5 Hz) in sitsirikine (**5**) while, respectively, at *δ*_H_ 3.92 (*J* = 11.0, 8.0 Hz) and 3.71 (*J* = 11.0, 3.5 Hz) in 16-*epi*-sitsirikine [20]. The configuration of C-16 in **3** was also kept *R* from the coupling constants of H-17a [*δ*_H_ 3.92 (dd, 11.0, 8.4 Hz)] and H-17b [*δ*_H_ 3.52 (dd, 11.0, 6.3 Hz)] were similar to those of sitsirikine (**5**).

By structural analysis, we found only compounds **1** and **2** showed cytotoxicity in NSCs proliferation assay, while none of the other isolated tetracyclic yohimbine-type alkaloids (**3**–**5**, **16**–**17**, and **20**–**26**) (Fig. 1) was active at 10 μM, which suggested that the carboxylic acid moieties in **1** and **2** may be the key groups contributing to NSCs toxicity. Besides, the visible activities difference between **1** and **2** (Table 1) indicated that the oxidation of N_4_ may reduce the inhibitory effects of **2**,which also can be used to explain why compounds **6**–**7** exhibited inhibitory activities but their *N*_4_-oxide derivative (**11**–**12**) did not work. Interestingly, may due to the configuration change of C-7, compound **14** was not active, while its *N*_4_-oxide derivative (**10)** inhibited NSCs proliferation at tested concentration.

## Experimental Section

### Plant Material

The hook-bearing branches of *U. rhynchophylla* were purchased from the Luo-Si-Wan Chinese herbal medicine market, Kunming, Yunnan province, China, in April 2016, and identified by Dr. Zhang Jun, Kunming Plant Classification Biotechnology Co., Ltd. A voucher specimen (No. WEI_20160418) was deposited in the State Key Laboratory of Phytochemistry and Plant Resources in West China, Kunming Institute of Botany, Chinese Academy of Sciences.

### General Experimental Procedures

Optical rotations were performed on a JASCO P-1020 polarimeter. IR spectra were measured on a Bruker FT-IR Tensor 27 spectrometer with KBr pellets. UV spectra were obtained on Shimadzu UV-2401A spectrometer. 1D-NMR and 2D-NMR spectra were recorded on an AV-600 MHz or a Bruker DRX-400 MHz spectrometer. Coupling constants were expressed in Hz and chemical shifts were given on a ppm scale with tetramethylsilane as internal standard. HRESIMS were recorded on an API QSTAR Pulsar 1 spectrometer. CD spectra were obtained on a JASCO 810 spectrometer. Column chromatography (CC) was performed on silica gel (200–300 mesh, Qingdao Marine Chemical Ltd., Qingdao, People’s Republic of China), Sephadex LH-20 (Pharmacia Fine Chemical Co., Ltd., Sweden), and MCI-gel CHP 20P (75–100 μm, Mitsubishi Chemical Co., Ltd). Thin-layer chromatography (TLC) was carried out on silica gel H-precoated plates (Qingdao Haiyang Chemical Co., Ltd.) with CHCl_3_/MeOH (9:1, 4:1, v/v) as developing solvents and spots were visualized by Dragendorff’s reagent. High performance liquid chromatography (HPLC) was performed using waters 600 equipment with semi-preparative and preparative C_18_ columns (150 × 9.4 and 250 × 21.2 mm, respectively).

### Extraction and Isolation

The air-dried and powdered hook-bearing branches of *U. rhynchophylla* (10 kg) were extracted with MeOH (50 L × 3) under reflux conditions at 70 °C, 3 h for each time. After removal of the organic solvent under reduced pressure, the residue was dissolved in 0.3% aqueous hydrochloric acid (v/v); the solution was subsequently basified to pH 9–10 using ammonia and then extracted with EtOAc (3 L × 4) to give an alkaloidal extract. The extract (52 g) was subjected to a silica gel column (CHCl_3_/MeOH, 1:0–0:1) to afford fractions (A-F). Fr. A (4.8 g) was subjected to silica gel column chromatography (CC) using a petroleum ether/acetone gradient (10:1–1:9) to afford sub-fractions (Fr. 1–4). Fr. 3 (1.2 g) was further purified on Sephadex LH-20 using MeOH under isocratic conditions to afford isocorynoxeine (**6**) (12 mg), augustine (**29**) (12 mg), and corynoxeine (**13**) (8 mg). Fr. 4 (1.5 g) purified on Sephadex LH-20 column by isocratic elution with MeOH to get a mixture (400 mg), which was then further separated on a semi-preparative C_18_ HPLC column with a gradient of MeOH/H_2_O (1:1–1:0) to obtain isorhynchophylline (**7**) (5 mg) and rhynchophylline (**14**) (6 mg). Fr. B (9.8 g) was chromatographed on silica gel column (CHCl_3_/MeOH, 9:1–0:1) to yield seven fractions (Fr. 5–11). Fr. 6 (0.8 g) was purified on Sephadex LH-20 column with MeOH under isocratic elution and further purified on a semi-preparative C_18_ HPLC column (MeOH/H_2_O, 1:1–1:0, v/v) to afford corynantheine (**20**) (12 mg), dihydrocorynantheine (**21**) (6 mg), sitsirikine (**5**) (11 mg), and geissoschizine methyl ether (**4**) (5 mg). Fr. C (10 g) was fractionated on MCI-gel CHP 20P column by eluting with a gradient of MeOH (30–100%) in H_2_O to yield four fractions (Fr. 12–15). Fr. 14 (200 mg) was subjected to Sephadex LH-20 CC using MeOH under isocratic elution and was further purified on a semi-preparative C_18_ HPLC column (MeOH/H_2_O, 40:60–80:20, v/v) to yield akuammigine (**18**) (3 mg), 3-*epi*-geissoschizine methyl ether (**17**) (1.2 mg), hirsuteine (**22**) (7 mg), and nitrocadambine B (**28**) (9 mg). Fr. D (12 g) was subjected to silica gel CC using a CHCl_3_/MeOH gradient (9:1–0:1) to give six fractions (Fr. 16–21). Fr. 20 (800 mg) was subjected to MCI-gel CHP 20P column using MeOH/H_2_O gradient (3:7–1:0) and further separated on Sephadex LH-20 column using MeOH with isocratic elution to yield (4*S*)-akuammigine *N*-oxide (**8**) (2 mg) and hirsuteine *N*-oxide (**26**) (3 mg). Fr. 21 (2.5 g) was subjected to Sephadex LH-20 column using MeOH with isocratic elutionand was further purified on a semi-preparative C_18_ HPLC column (MeOH/H_2_O, 40:60–80:20, v/v) to obtain 3*α*-dihydrocadambine (**27**) (8 mg), (4*S*)-rhynchophylline *N*-oxide (**10**) (7 mg), and (4*S*)-corynoxeine *N*-oxide (**15**) (6 mg). Fr. E (7 g) was chromatographed over silica gel column using a CHCl_3_/MeOH gradient (4:1–0:1) to obtain seven fractions (Fr. 22–28). Fr. 24 (200 mg) was further subjected to Sephadex LH-20 column using MeOH to yield geissoschizine methyl ether *N*_4_-oxide (**16**) (10 mg) and hirsutine *N*-oxide (**23**) (9 mg). Fr. 25 (400 mg) was further purified on MCI-gel CHP 20P column with MeOH/H_2_O gradient (1:4–1:0) to afford (4*R*)-akuammigine *N*-oxide (**19**) (17 mg) and hirsutine (**24**) (13 mg). Fr. 26 (400 mg) was subjected to MCI-gel CHP 20P column using MeOH/H_2_O gradient (1:4–1:0) and was further purified on a semi-preparative C_18_ HPLC column (MeOH/H_2_O, 40:60–80:20, v/v) to yield dihydrocorynantheine *N*-oxide (**25**) (18 mg) and 3*β*-sitsirikine *N*_4_-oxide (**3**) (7 mg). Fr. 27 (300 mg) was repeatedly chromatographed over Sephadex LH-20 column with MeOH to afford isorhynchophylline *N*_4_-oxide (**11**) (5 mg), (4*S*)-isocorynoxeine *N*-oxide (**12**) (4 mg), and cadambine (**9**) (10 mg). Fr. 28 (300 mg) was subjected to MCI-gel CHP 20P column using MeOH/H_2_O gradient (1:4–1:0) and was further purified on a semi-preparative C_18_ HPLC column (MeOH/H_2_O, 30:70–80:20, v/v) to yield geissoschizic acid (**1**) (20 mg) and geissoschizic acid *N*_4_-oxide (**2**) (2 mg).

*Geissoschizic acid* **(1)**: colorless amorphous solid; [α]_D_^26^ +31.9 (*c* 0.09, MeOH); UV (MeOH) *λ*_max_ (log *ε*): 269 (4.05), 223 (4.74), 207 (4.60) nm; ECD (*c* 0.15 mM, MeOH) λ (Δ*ε*): 218 (−7.19), 234 (+3.71), 267 (+2.48); IR (KBr) *ν*_max_ 3421, 2933, 1644, 1382, 1238, and 1132 cm^−1^; HRESIMS *m/z* 353.1852 [M + H]^+^ (calcd for C_21_H_25_N_2_O_3_, 353.1860); ^1^H and ^13^C NMR data, see Table 2.

*Geissoschizic acid N*_4_-*oxide* **(2)**: colorless amorphous solid; [α]_D_^21^ +101.8 (*c* 0.11, MeOH); UV (MeOH) *λ*_max_ (log *ε*): 269 (3.42), 223 (4.11), 208 (4.00) nm; ECD (*c* 0.61 mM, MeOH) λ (Δ*ε*): 219 (−8.02), 231 (+4.21), 266 (+2.03); IR (KBr) *ν*_max_ 3393, 2943, 1636, 1448, 1246, and 1113 cm^−1^; HRESIMS *m/z* 369.1806 [M+H]^+^ (calcd for C_21_H_25_N_2_O_4_, 369.1809); ^1^H and ^13^C NMR data, see Table 2.

*3β*-*sitsirikine N*_*4*_-*oxide* **(3)**: colorless amorphous solid; [α]_D_^24^ +104.3 (*c* 0.04, MeOH); UV (MeOH) *λ*_max_ (log *ε*): 273 (4.04), 221 (4.72) nm; ECD (*c* 0.13 mM, MeOH) λ (Δ*ε*): 213 (+7.41), 234 (+1.88), 262 (−2.07); IR (KBr) *ν*_max_ 3414, 2930, 1727, 1633, 1454, 1319, and 1063 cm^−1^; HRESIMS *m/z* 371.1960 [M+H]^+^ (calcd for C_21_H_27_N_2_O_4_, 371.1965); ^1^H and ^13^C NMR data, see Table 2.

### NSCs Proliferation Assay

Neural stem cells (NSCs) were grown in serum-free growth medium (DMEM/F12 1:1; Hyclone) containing 20 ng/mL human epidermal growth factor (EGF, Gibco), 20 ng/mL human fibroblast growth factor (bFGF, Gibco), 1% penicillin/streptavidin, 1% N_2_ supplement (Gibco), 1 × B27 (Gibco) and 10 μg/mL heparin as previously described [38–39]. The media were allowed to change every 2 days. The resulting neurospheres were passaged every 3–4 days to single-cell suspension for continued growth and expansion of stem cells. For treatment experiments, NSCs were treated with 0.1% DMSO, indicated compounds (10 μM), and puromycin (positive control) (10 μM).

Cell clusters generated by adult neural stem cells (NSCs) proliferation were trypsinized into single cell and evenly plated into 96 well plate overnight. NSCs proliferation rate was measured by SRB assay according to the standard protocol [40]. Briefly, cells were exposed to DMSO and the test compounds and incubated at 37 °C in a humidified incubator with 5% CO_2_ for 48 h, without removing the cell culture supernatant, cells were fixed with 16% TCA and incubated at 4 °C for 1 h. Plates were washed 5 times with water and air dried. A 100 μL of sulforhodamine B solution 0.4% (w/v) in 1% acetic acid was added into each well. After 10 min at room temperature, the plates were rinsed five times with 1% acetic acid quickly to remove unbound dye and then air dried. Then 50 μL 10 mM un-buffered Tris-base was added to solubilize the protein-bound dye. The absorbance was read on automated plate reader (Epoch, Biotek) at 515 nm. Each compound was treated in 3 independent wells per experiment, and the assay was repeated at least 3 times. The value of DMSO was set to 1, and the other values were normalized to DMSO.

## Electronic supplementary material

Below is the link to the electronic supplementary material.

1D and 2D NMR spectra, ECD, HRESIMS, and UV spectra of compounds **1–3** are available as Supplementary Information. Supplementary material 1 (PDF 929 kb)
