# Supplementary material for: Indole Alkaloids Inhibiting Neural Stem Cell from Uncaria rhynchophylla
Source: Nat Prod Bioprospect. 2017 Sep 26;7(5):413–9. doi: 10.1007/s13659-017-0141-y (PMC5655363; doi:10.1007/s13659-017-0141-y)
Supplement: Supplementary file 1 — 1D and 2D NMR spectra, ECD, HRESIMS, and UV spectra of compounds 1–3 are available as Supplementary Information. Supplementary material 1 (PDF 929 kb) [file 13659_2017_141_MOESM1_ESM.pdf]

Supporting Information

Indole Alkaloids Inhibiting Neural Stem Cell from Uncaria rhynchophylla

Xin Wei[a,d], Li-Ping Jiang[b,d], Ying Guo[a,d], Afsar Khan[c], Ya-Ping Liu[a], Hao-Fei Yu[a,d], Bei Wang[a,d], Cai-Feng Ding[a,d], Pei-Feng Zhu[a,d], Ying-Ying Chen[a], Yun-Li Zhao[a], Yong-Bing Chen[b], Yi-Fen Wang[a*], Xiao-Dong Luo[a,e,*]

[a]*State Key Laboratory of Phytochemistry and Plant Resources in West China, Kunming Institute of Botany, Chinese Academy of Sciences, Kunming 650201, People's Republic of China*

[b]*Key Laboratory of Animal Models and Human Disease Mechanisms, Kunming Institute of Zoology, Chinese Academy of Sciences, Kunming 650223, People's Republic of China*

[c]*Department of Chemistry, COMSATS Institute of Information Technology, Abbottabad 22060, Pakistan*

[d]*University of Chinese Academy of Sciences, Beijing 100049, People's Republic of China*

[e]*Yunnan Key Laboratory of Natural Medicinal Chemistry, Kunming, 650201, P. R. China*

---

* **Corresponding author** Prof. Dr. Xiao-Dong Luo or Dr. Yi-Fen Wang, Kunming Institute of Botany, Chinese Academy of Sciences, Kunming 650201, P. R. China. E-mail: xdluo@mail.kib.ac.cn or wangyifen@mail.kib.ac.cn, Phone: +86-871-6522-3177

Contents

Fig. 1S. ^{1}H NMR spectrum of compound **1**

Fig. 2S. ^{13}C NMR spectrum of compound **1**

Fig. 3S. HSQC spectrum of compound **1**

Fig. 4S. HMBC spectrum of compound **1**

Fig. 5S. COSY spectrum of compound **1**

Fig. 6S. ROESY spectrum of compound **1**

Fig. 7S. ECD spectrum of compound **1**

Fig. 8S. HRESIMS spectrum of compound **1**

Fig. 9S. UV spectrum of compound **1**

Fig. 10S. ^{1}H NMR spectrum of compound **2**

Fig. 11S. ^{13}C NMR spectrum of compound **2**

Fig. 12S. HSQC spectrum of compound **2**

Fig. 13S. HMBC spectrum of compound **2**

Fig. 14S. COSY spectrum of compound **2**

Fig. 15S. ROESY spectrum of compound **2**

Fig. 16S. ECD spectrum of compound **2**

Fig. 17S. HRESIMS spectrum of compound **2**

Fig. 18S. UV spectrum of compound **2**

Fig. 19S. ^{1}H NMR spectrum of compound **3**

Fig. 20S. ^{13}C NMR spectrum of compound **3**

Fig. 21S. HSQC spectrum of compound **3**

Fig. 22S. HMBC spectrum of compound **3**

Fig. 23S. COSY spectrum of compound **3**

Fig. 24S. ROESY spectrum of compound **3**

Fig. 25S. ECD spectrum of compound **3**

Fig. 26S. HRESIMS spectrum of compound **3**

Fig. 27S. UV spectrum of compound **3**

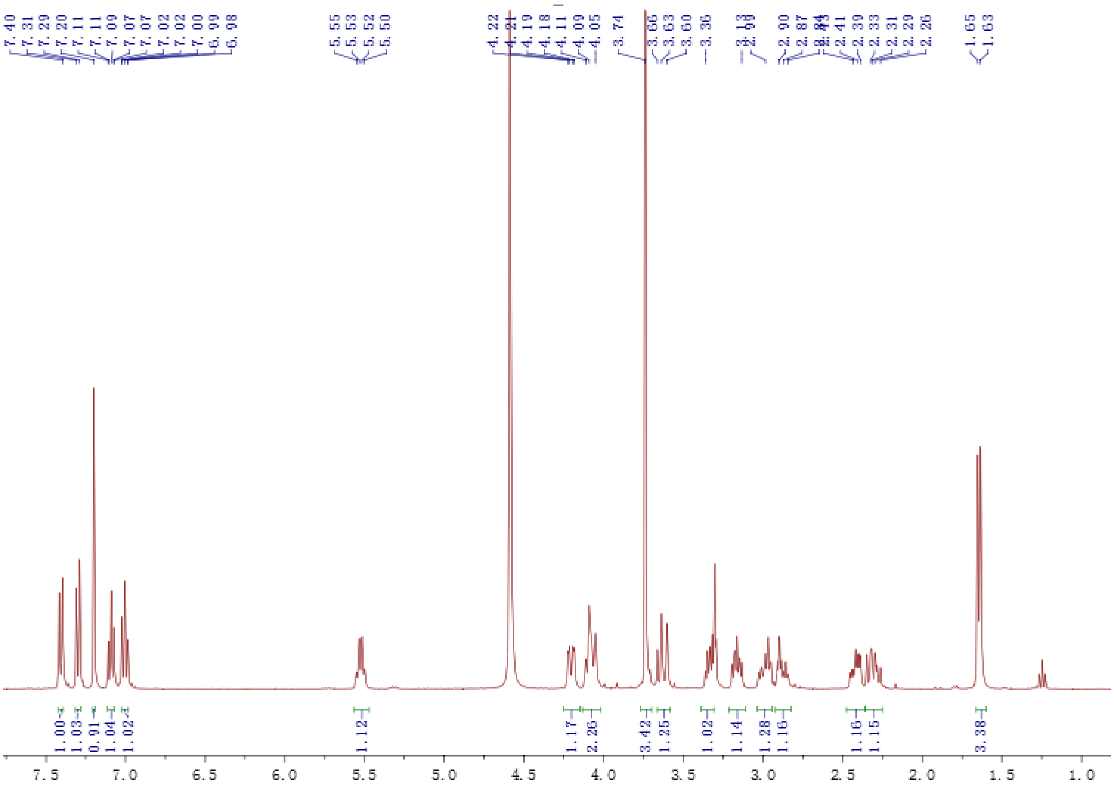

Fig. 1S ^{1}H NMR spectrum of compound **1** (CD_3OD, 400MHz)

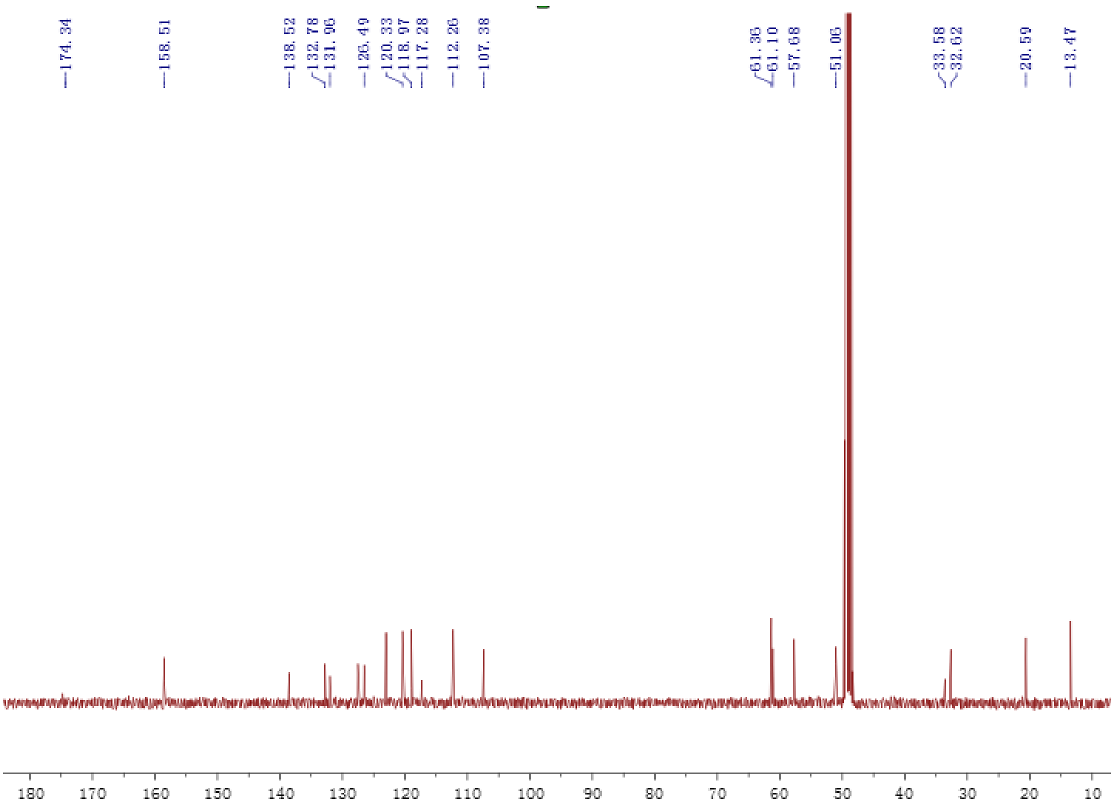

Fig. 2S ^{13}C NMR spectrum of compound **1** (CD_3OD, 100MHz)

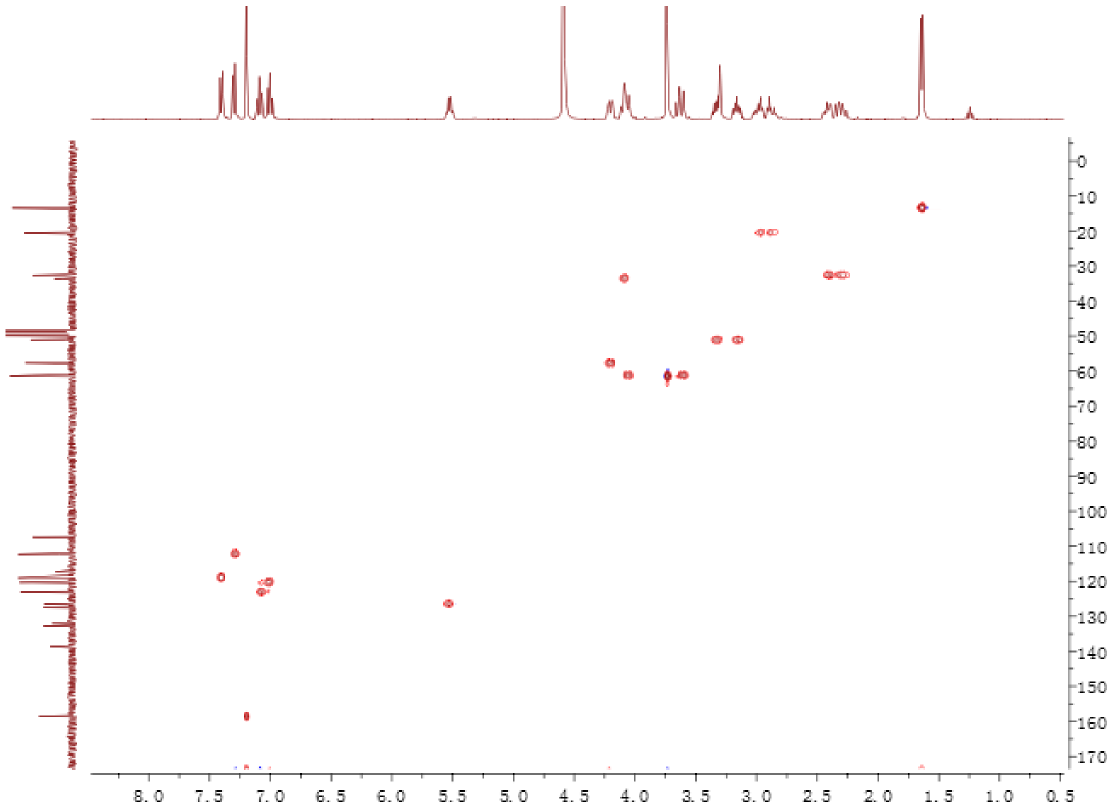

Fig. 3S HSQC spectrum of compound **1** (CD_3OD, 400MHz)

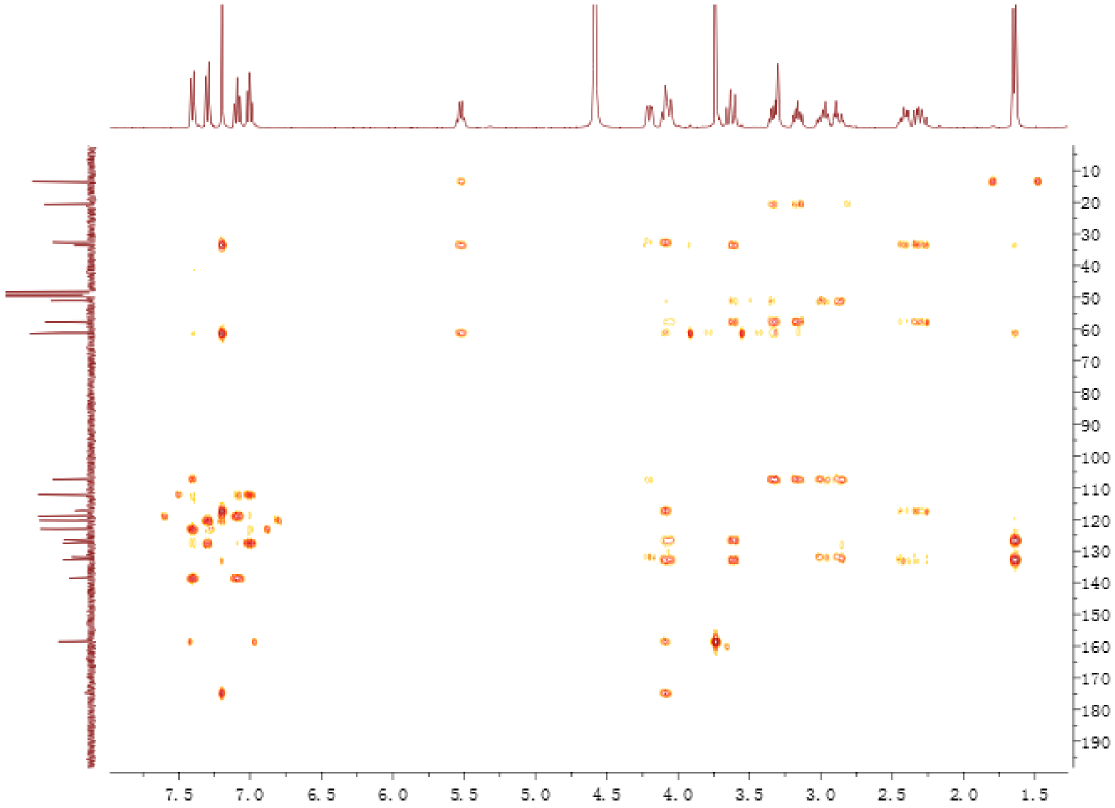

Fig. 4S HMBC spectrum of compound **1** (CD_3OD, 400MHz)

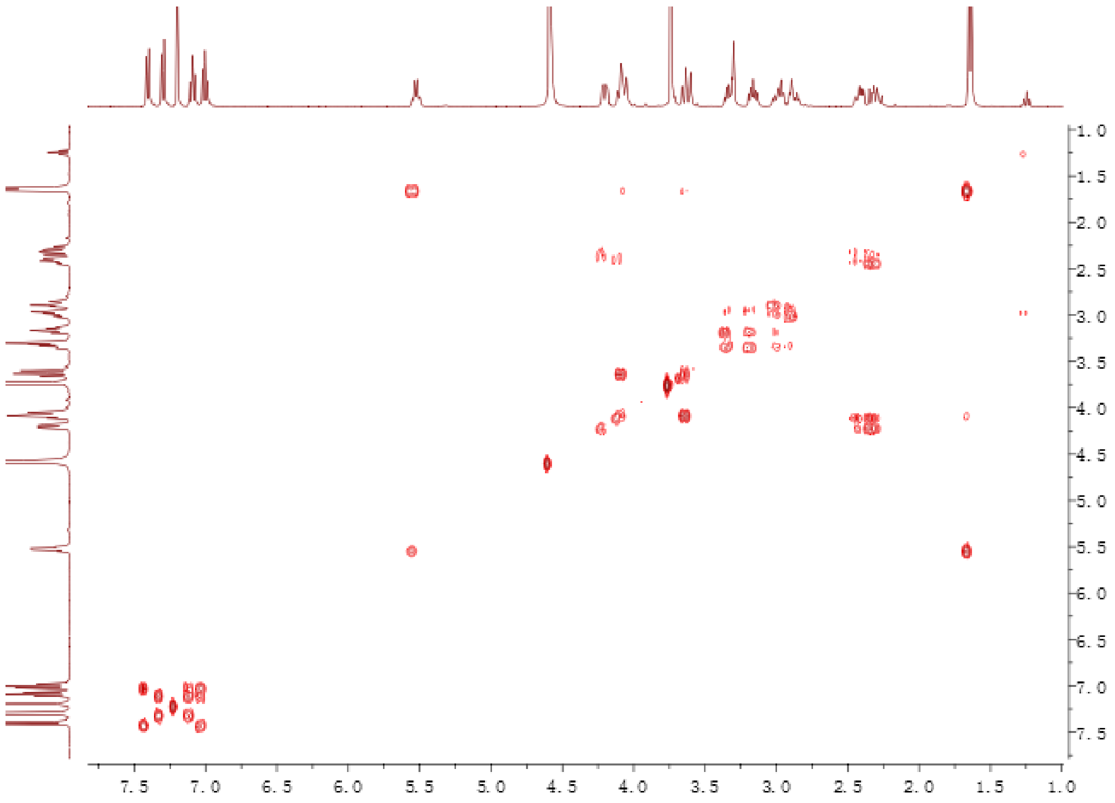

Fig. 5S COSY spectrum of compound **1** (CD_3OD, 400MHz)

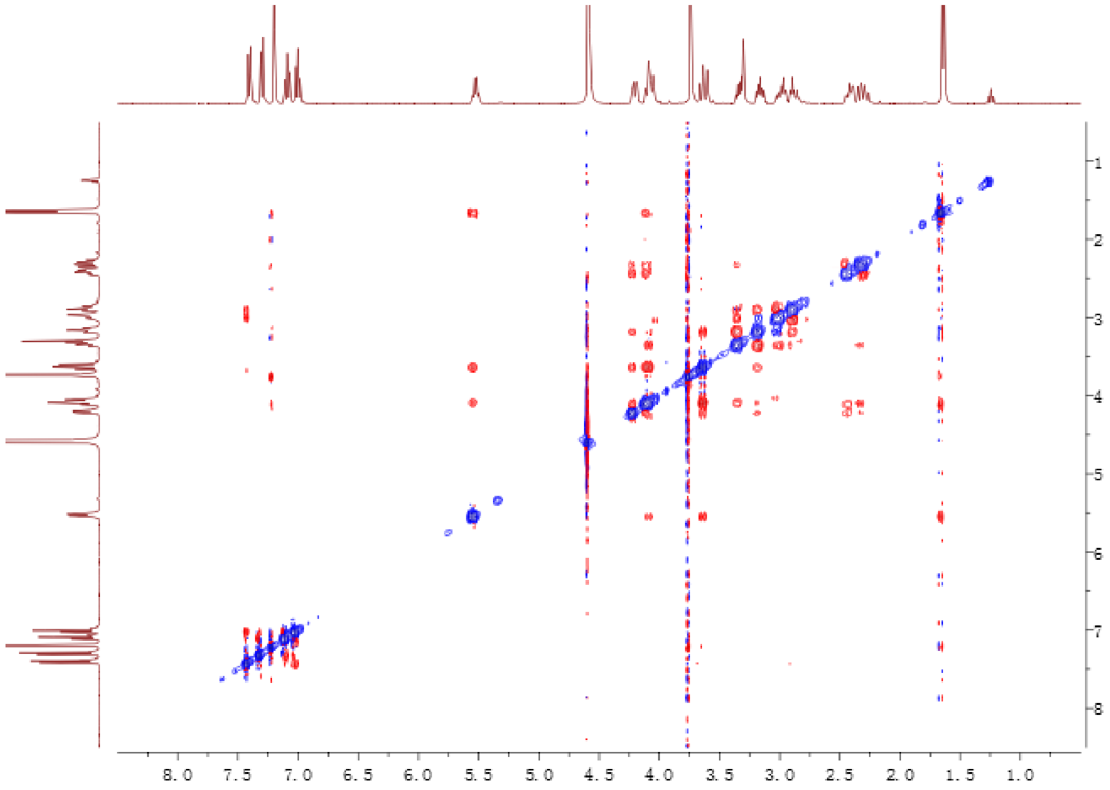

Fig. 6S ROESY spectrum of compound **1** (CD_3OD, 400MHz)

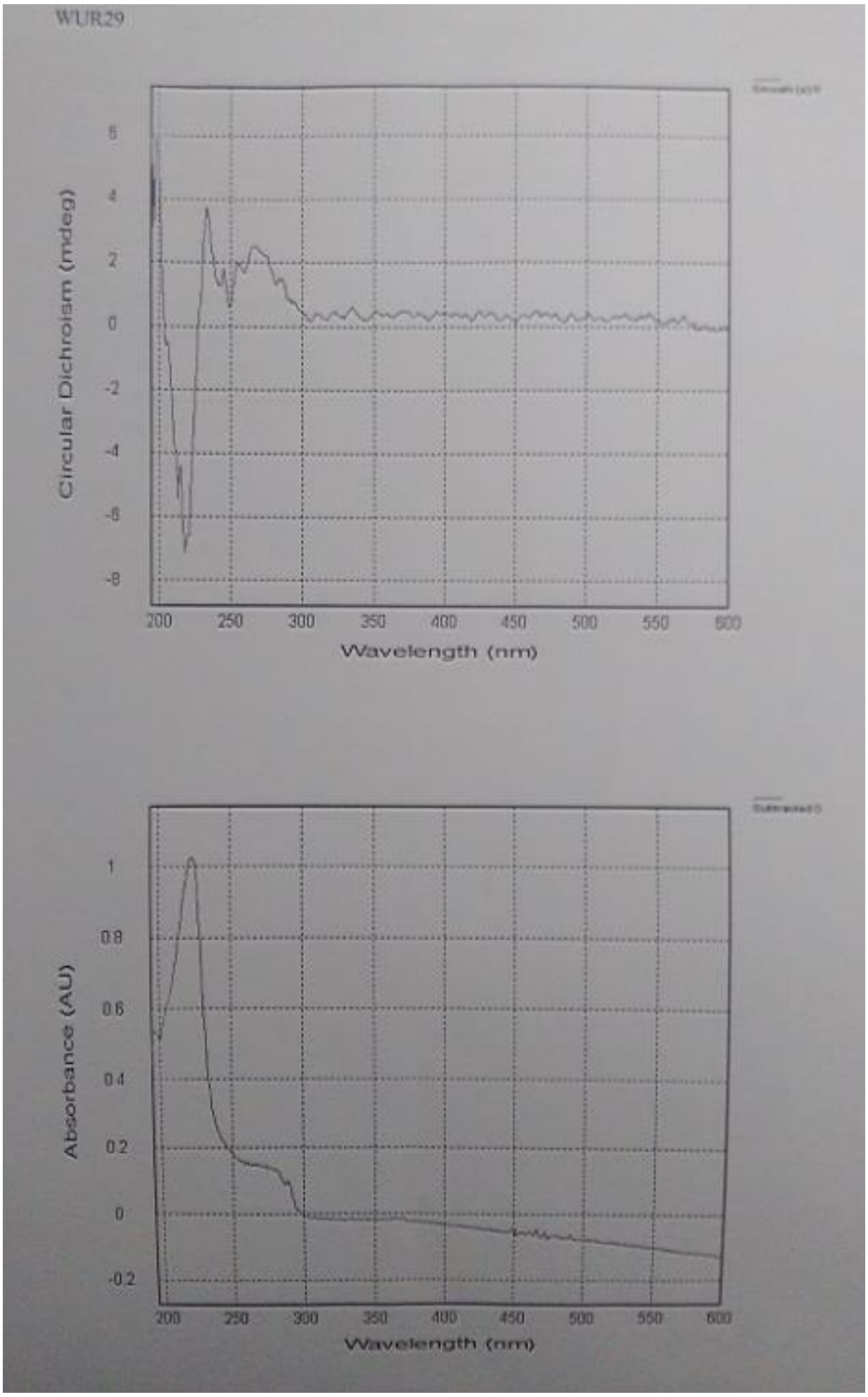

Fig. 7SECD spectrum of compound **1**

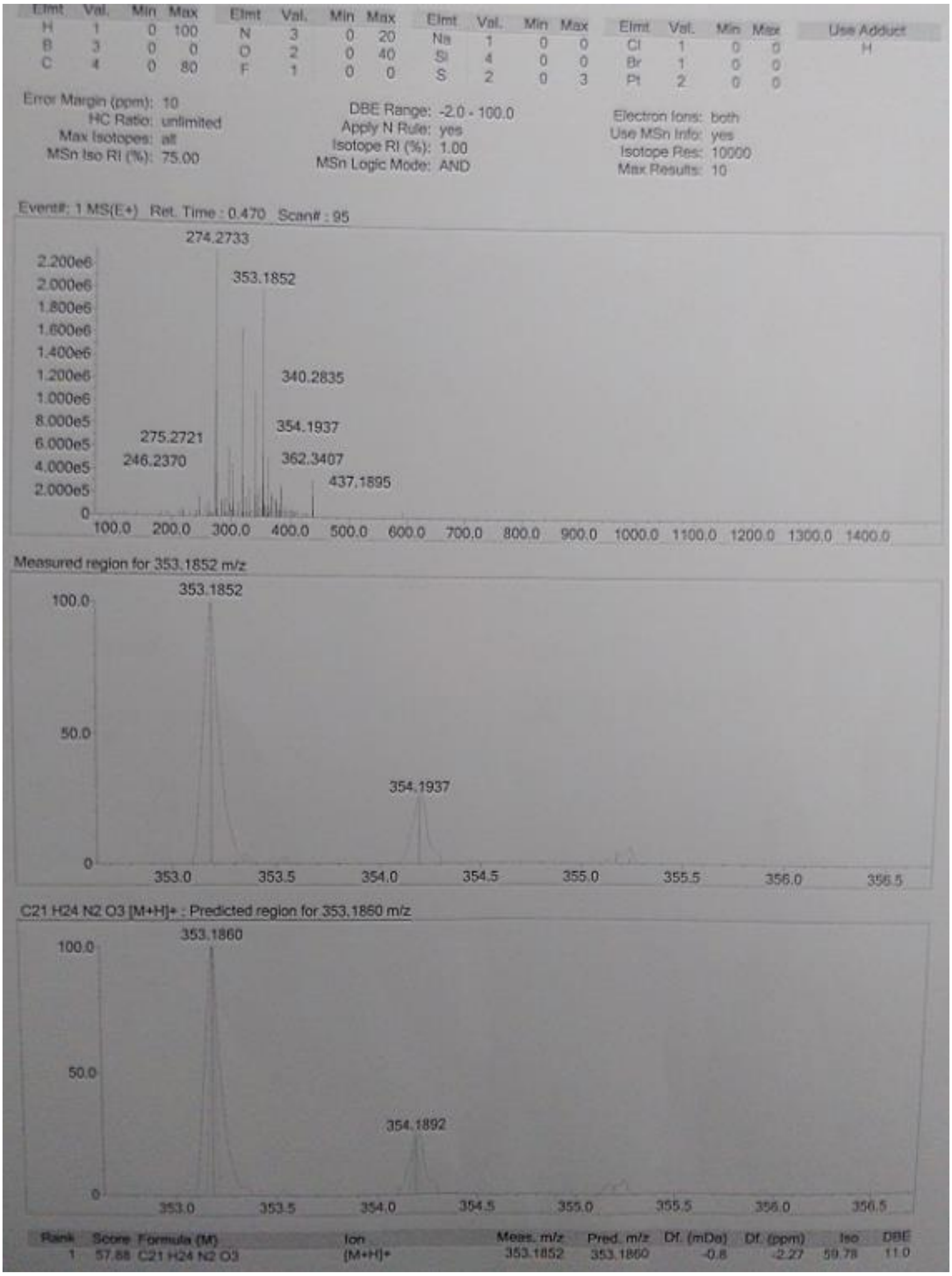

Fig. 8S HRESIMS spectrum of compound **1**

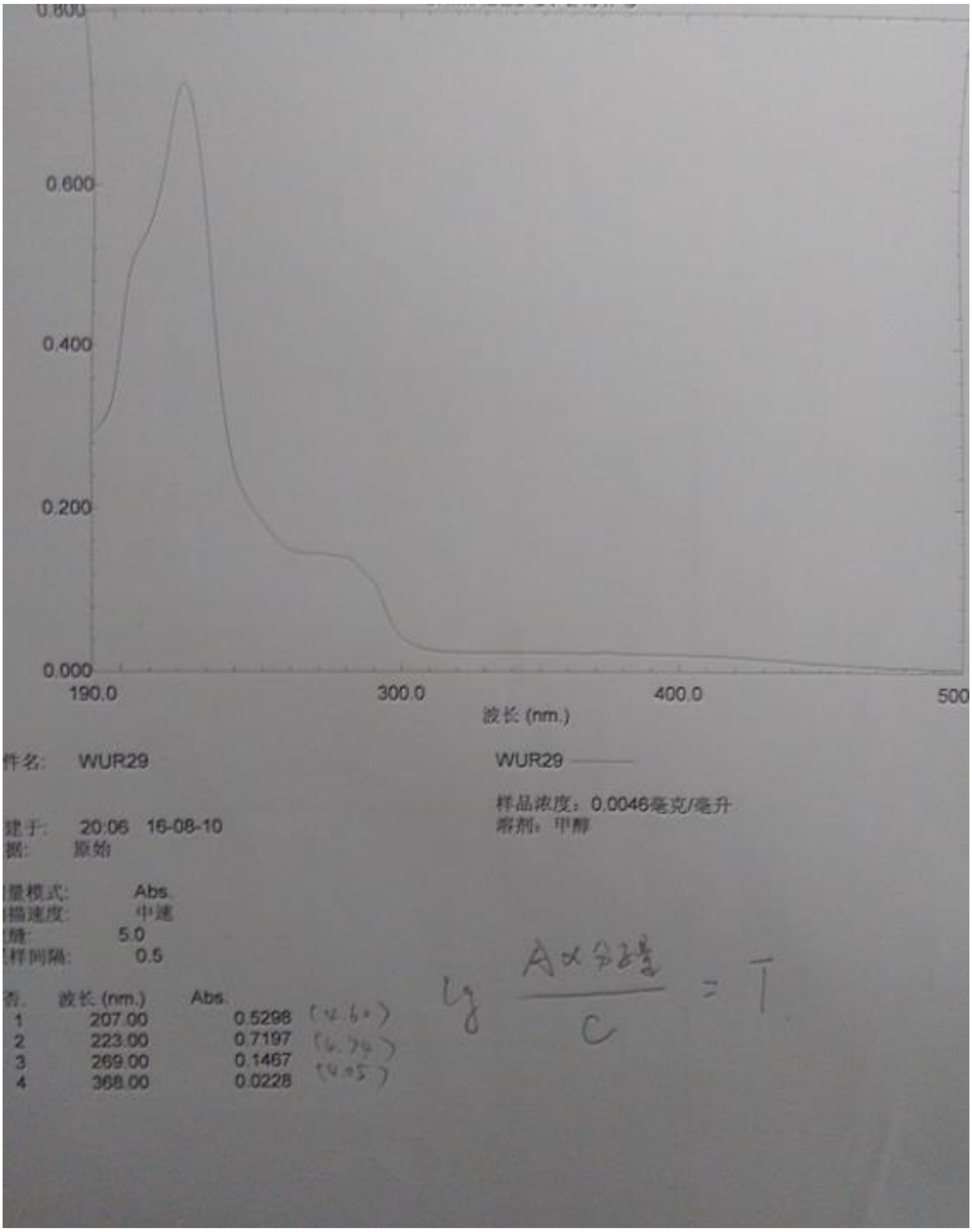

Fig. 9S UV spectrum of compound **1**

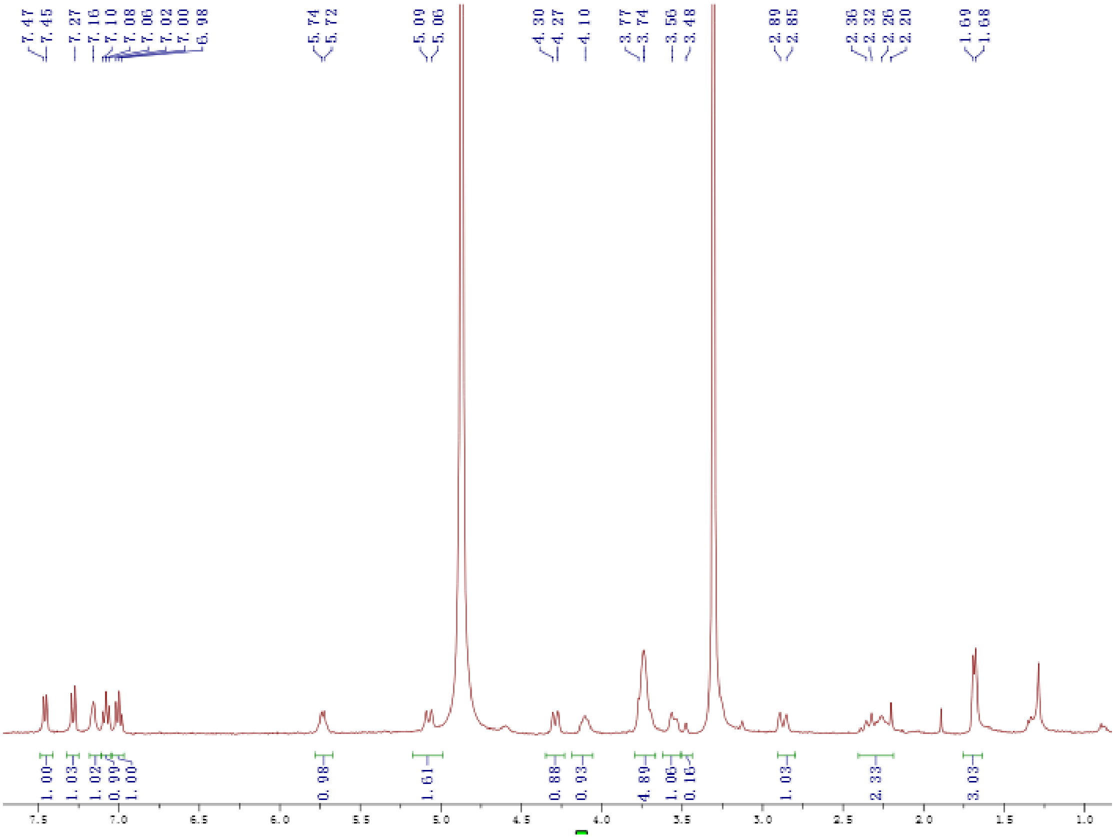

Fig. 10S ^{1}H NMR spectrum of compound **2**(CD_3OD, 400MHz)

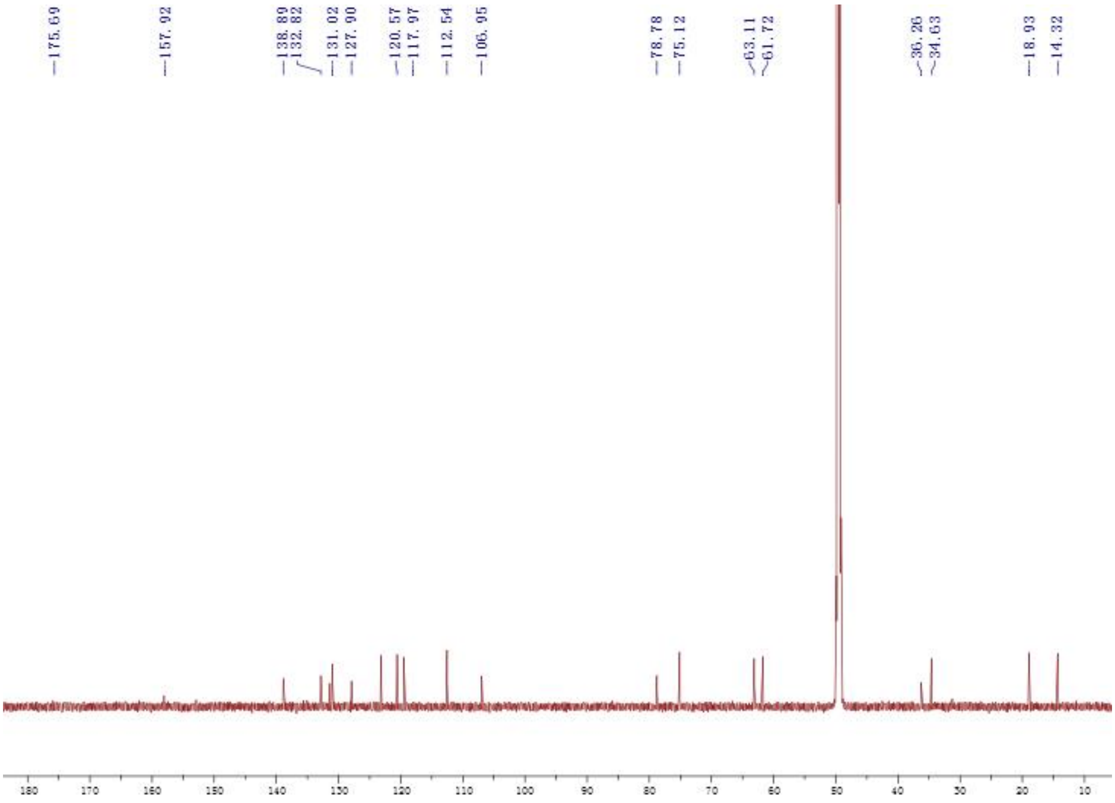

Fig. 11S ^{13}C NMR spectrum of compound **2**(CD_3OD, 150 MHz)

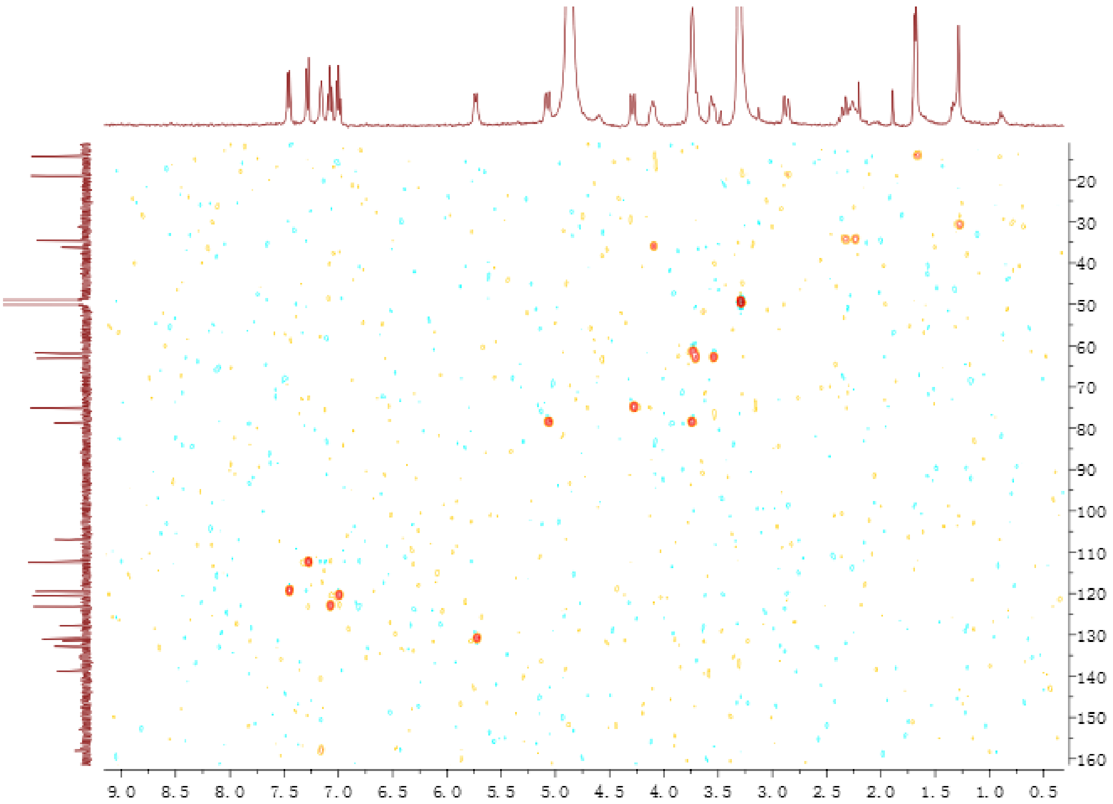

Fig. 12S HSQCspectrum of compound **2**(CD_3OD, 600 MHz)

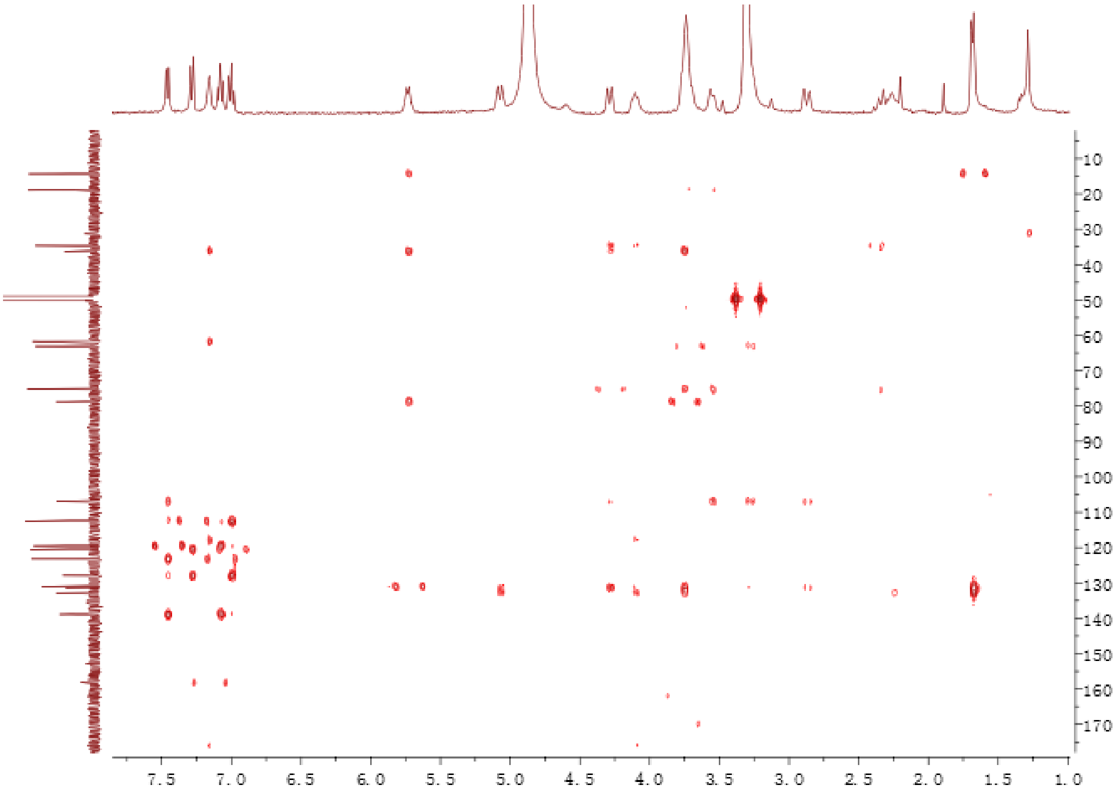

Fig. 13S HMBCspectrum of compound **2**(CD_3OD, 600 MHz)

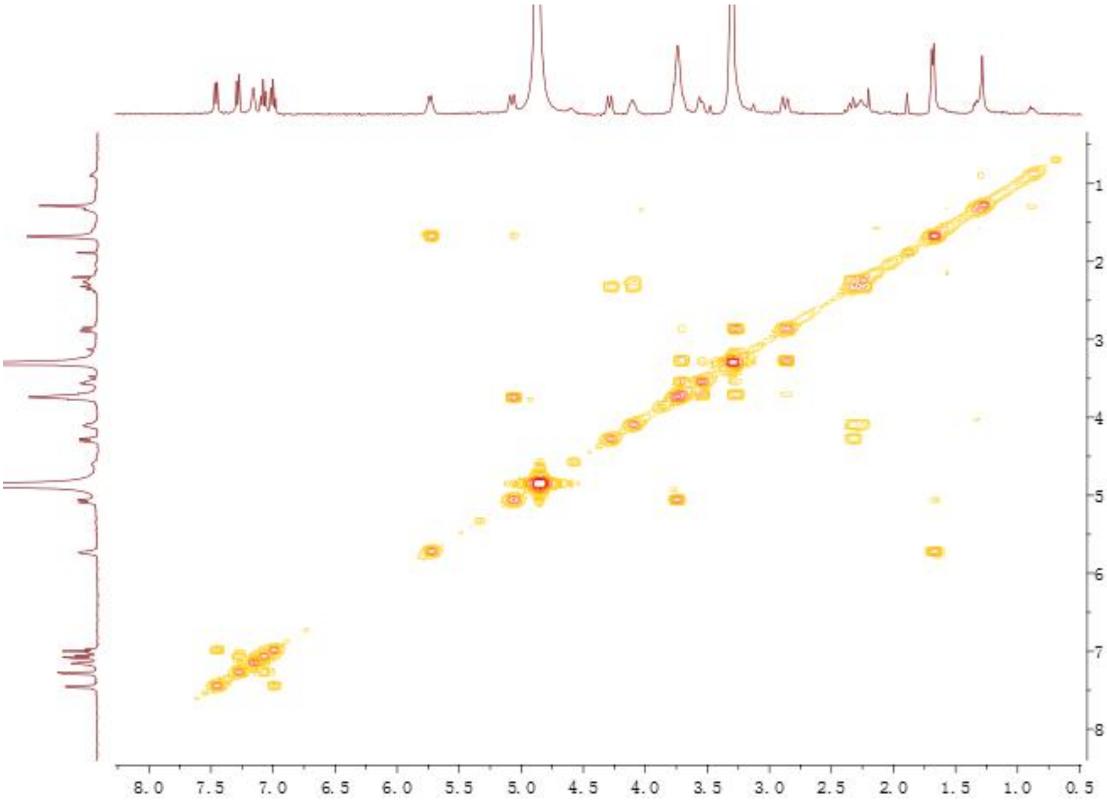

Fig. 14S COSYspectrum of compound **2**(CD_3OD, 600 MHz)

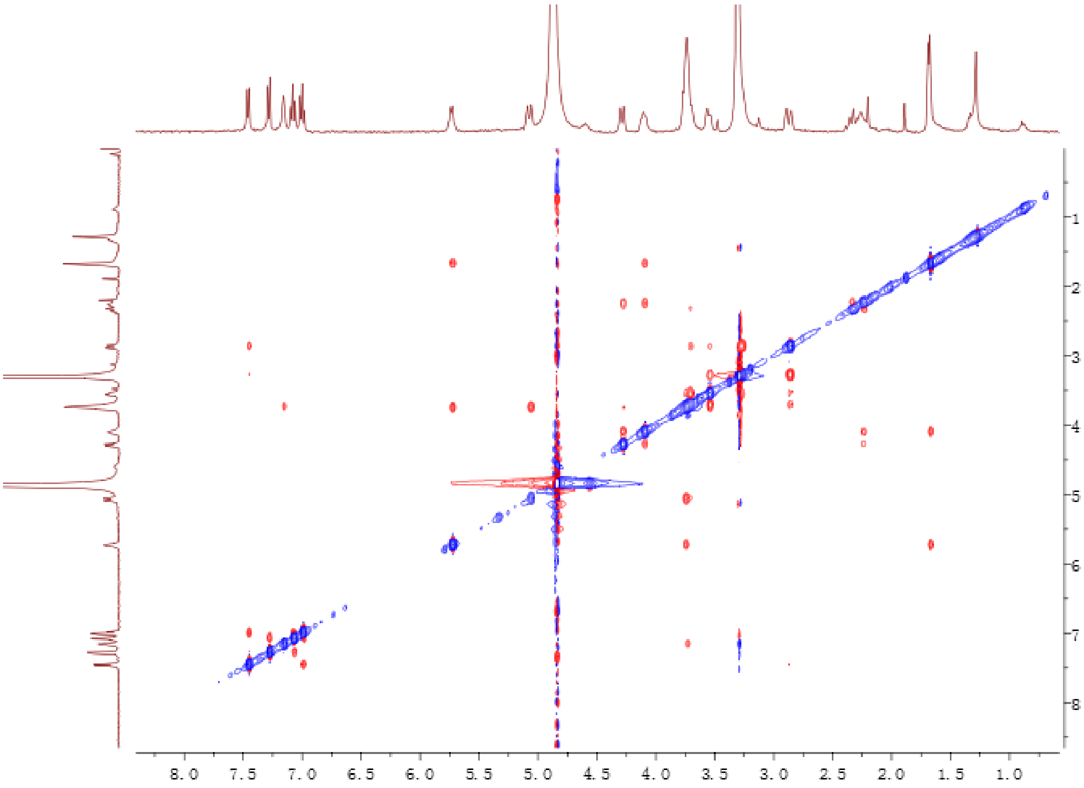

Fig. 15S ROESYspectrum of compound **2**(CD_3OD, 600 MHz)

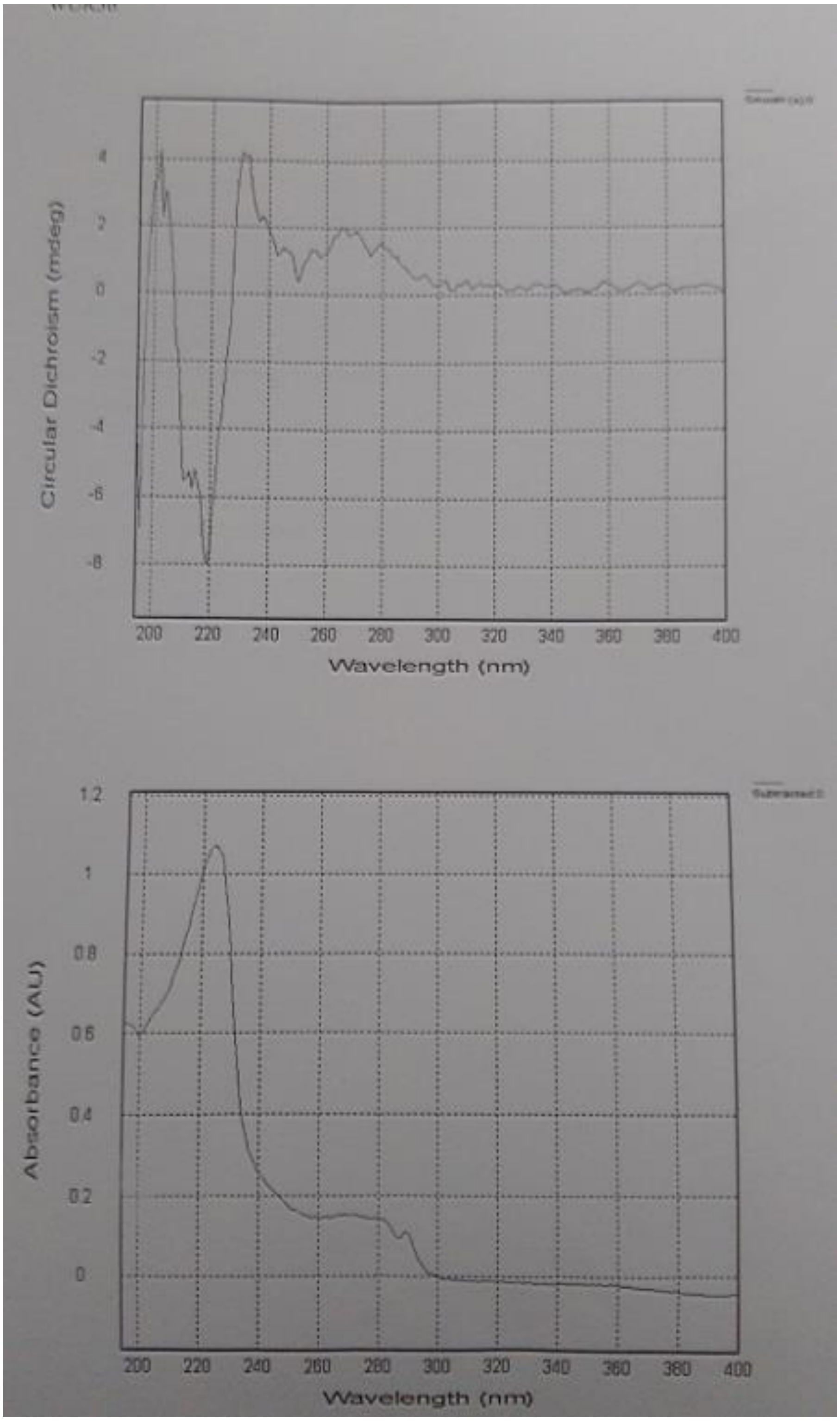

Fig. 16S ECD spectrum of compound **2**

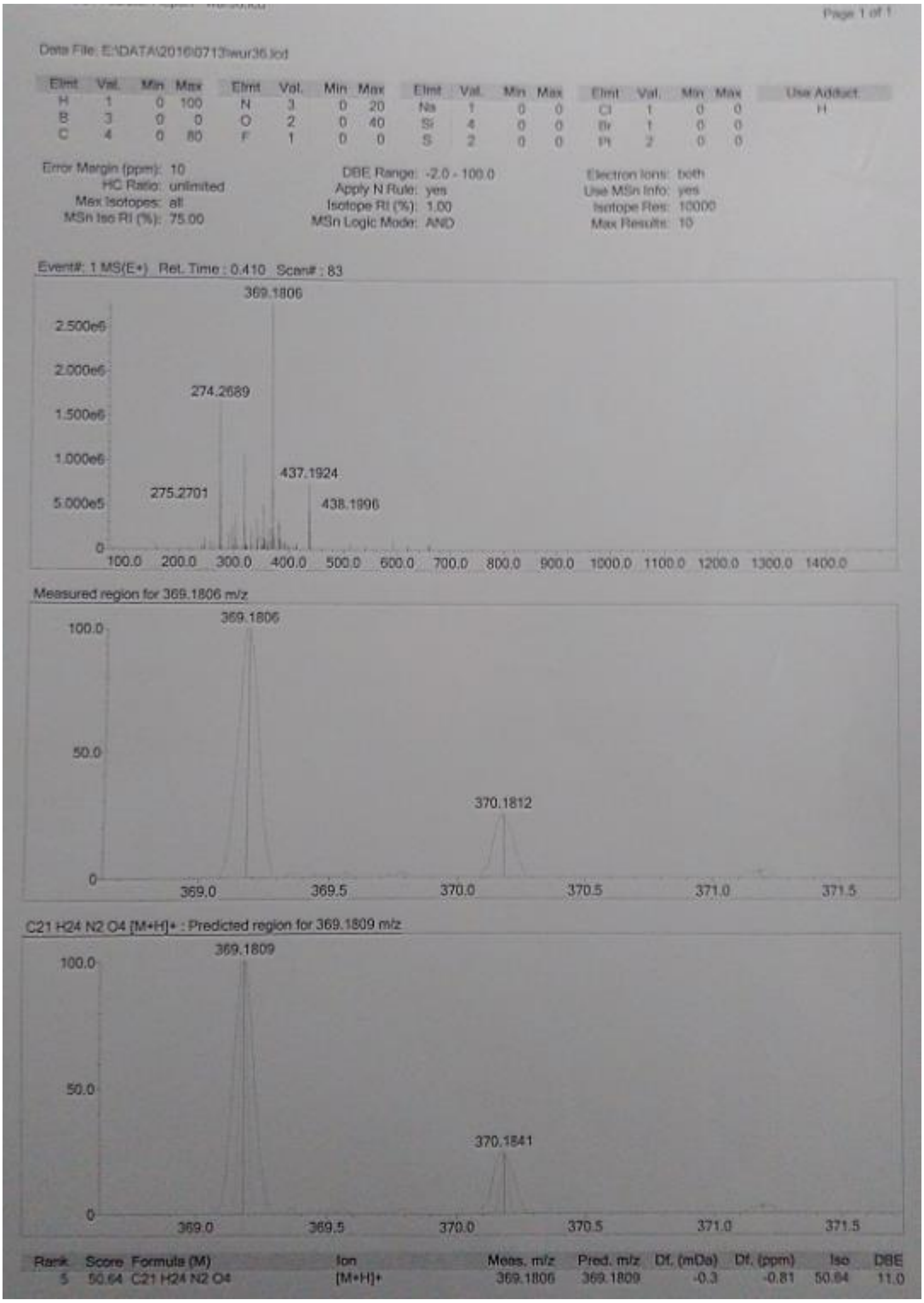

Fig. 17S HRESIMS spectrum of compound **2**

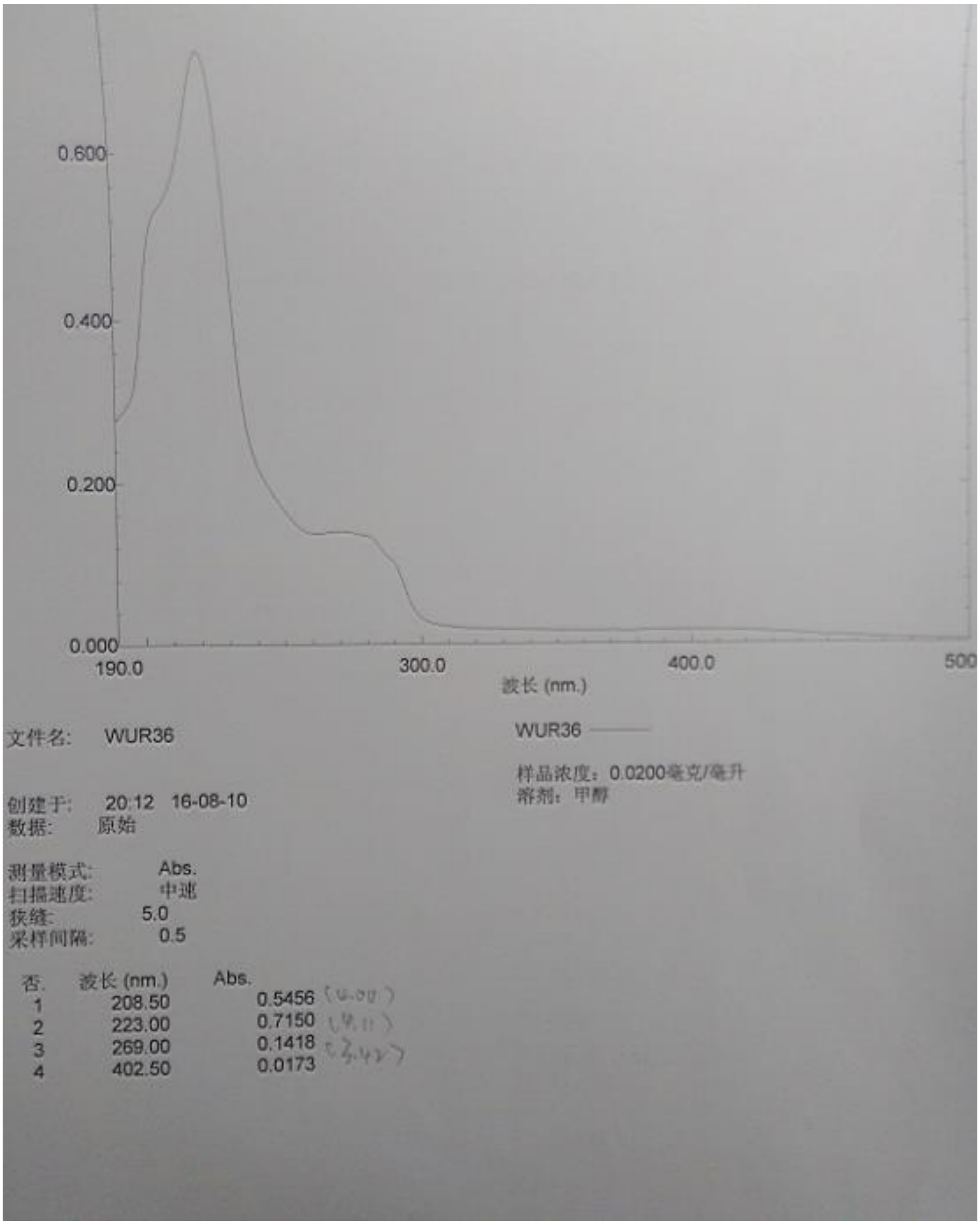

Fig. 18S UV spectrum of compound **2**

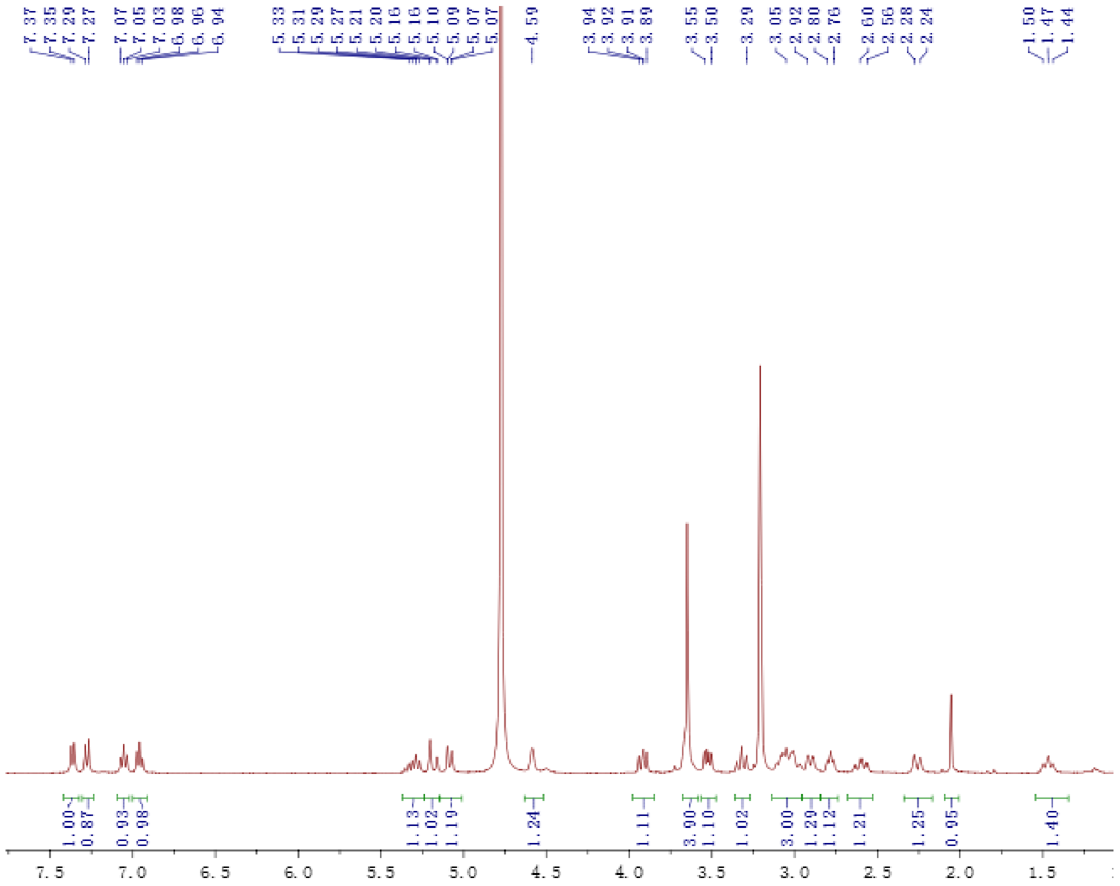

Fig. 19S ^{1}H NMR spectrum of compound **3**(CD_3OD, 600MHz)

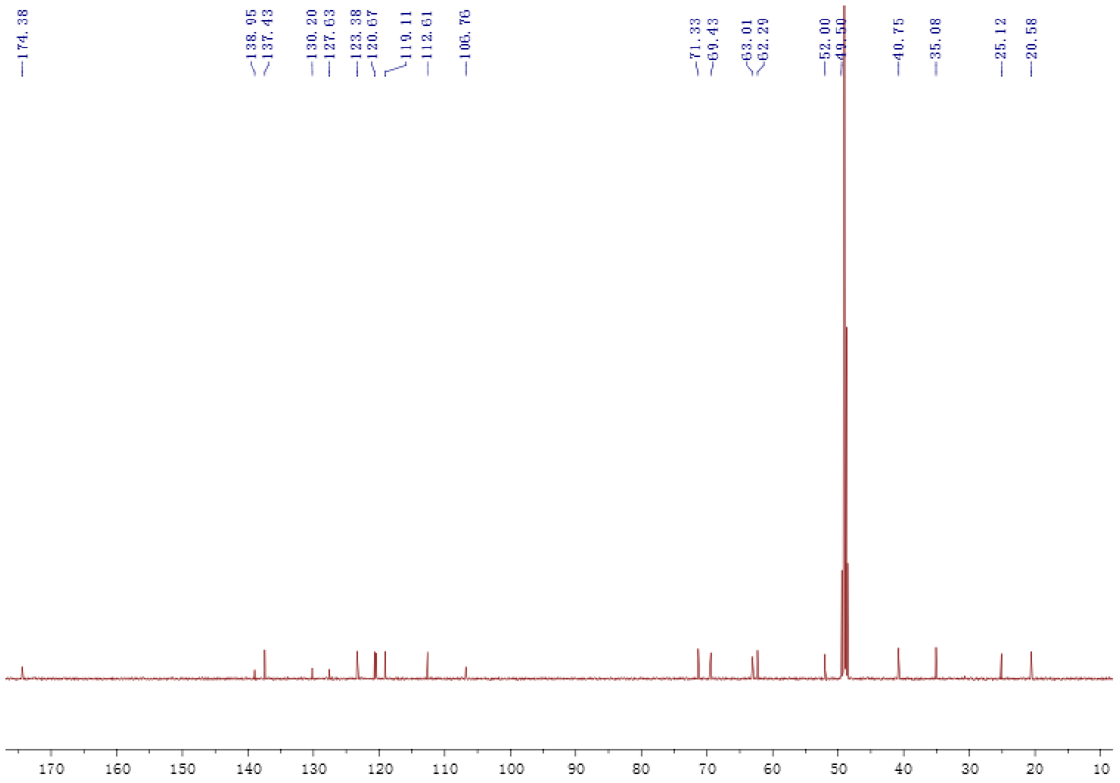

Fig. 20S ^{13}C NMR spectrum of compound **3**(CD_3OD, 150 MHz)

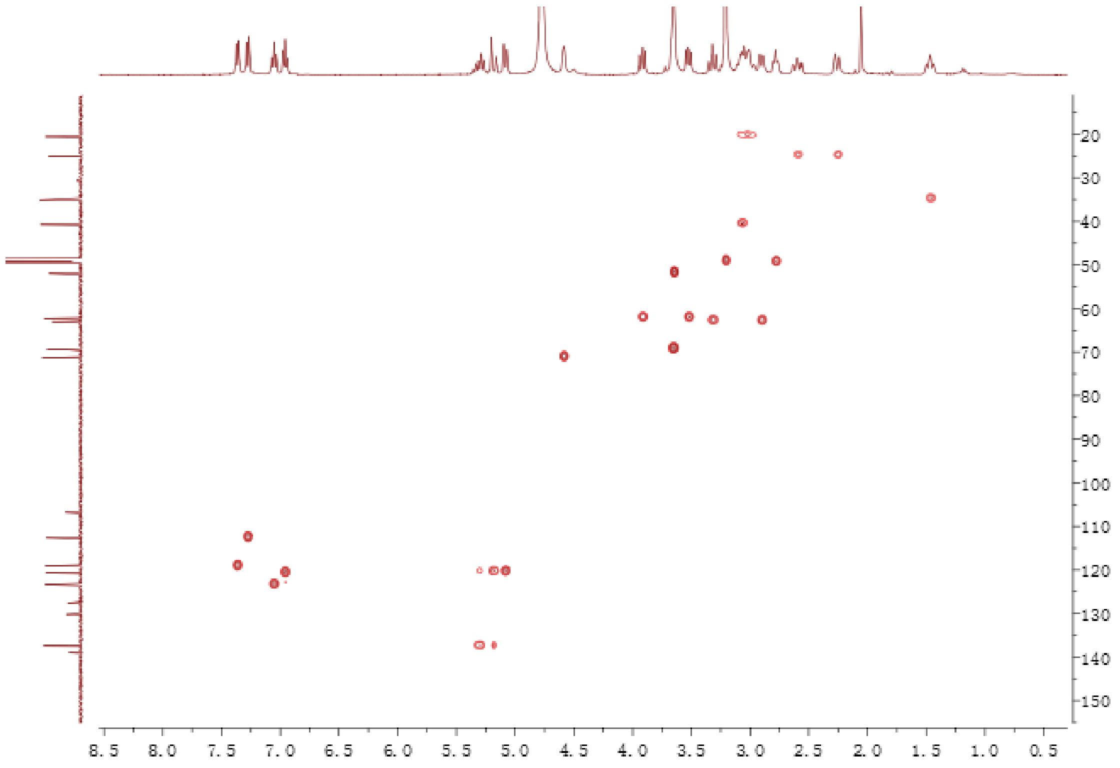

Fig. 21S HSQC spectrum of compound **3**(CD_3OD, 600MHz)\

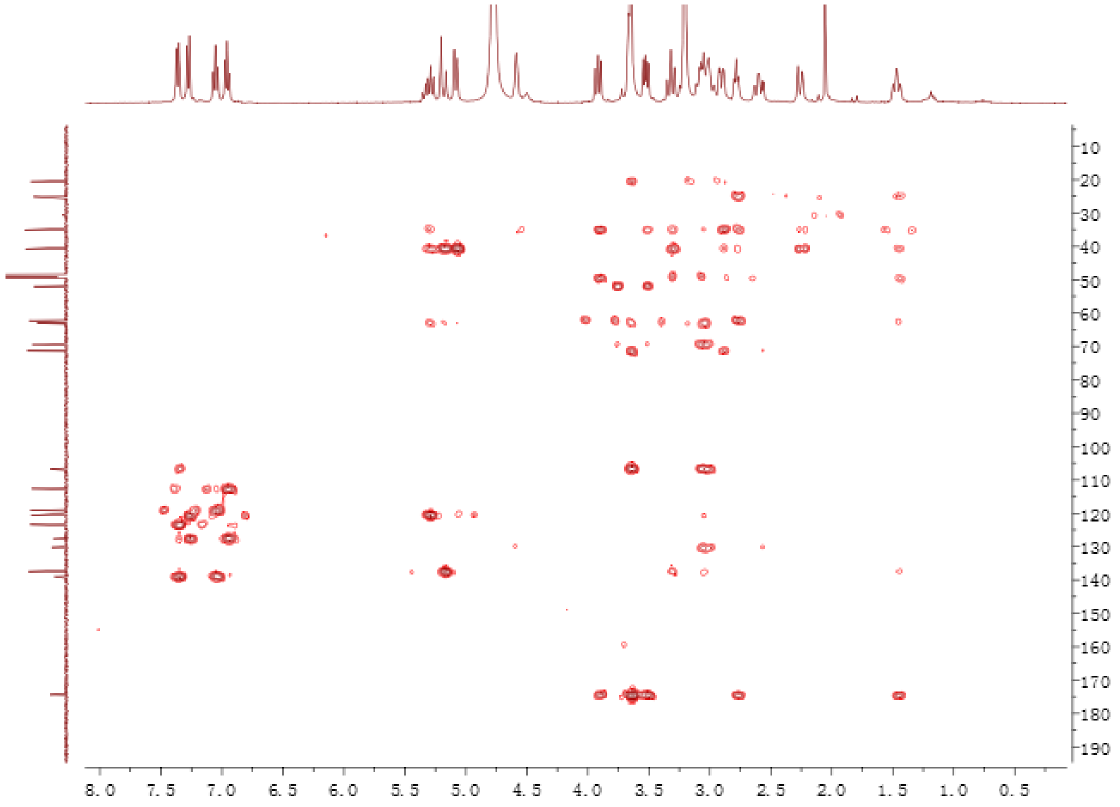

Fig. 22S HMBC spectrum of compound **3**(CD_3OD, 600MHz)

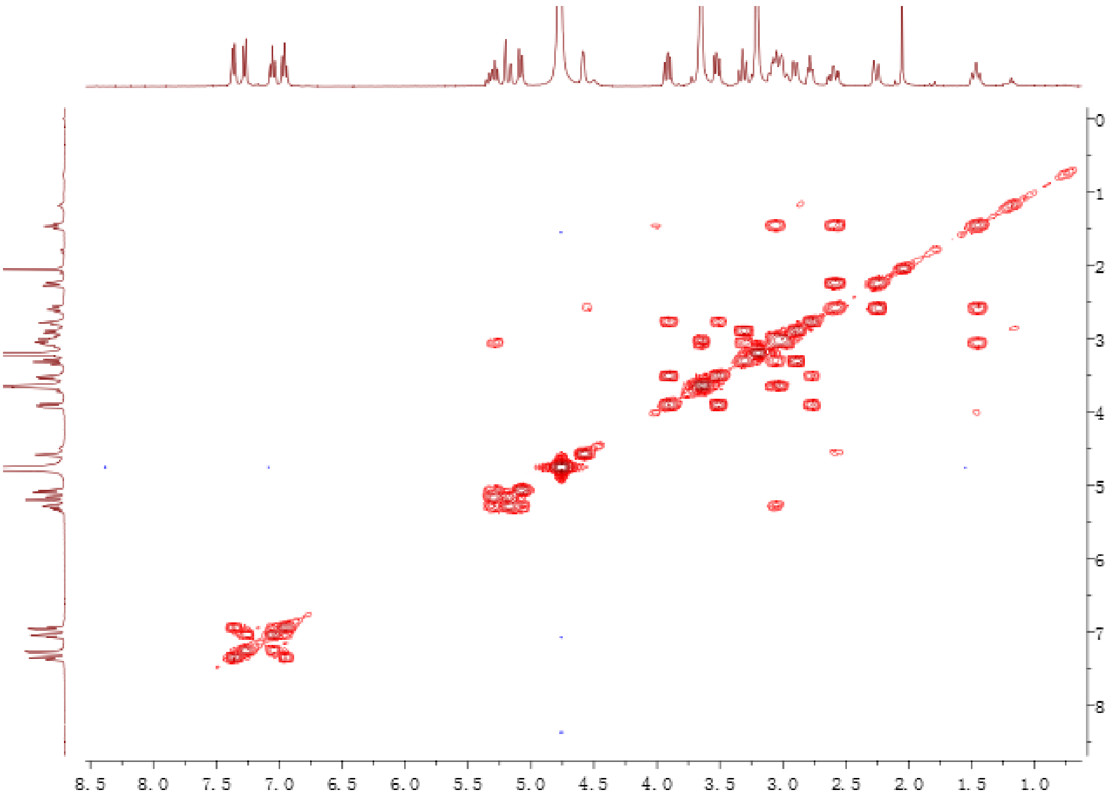

Fig. 23S COSY spectrum of compound **3**(CD$_3$OD, 600MHz)

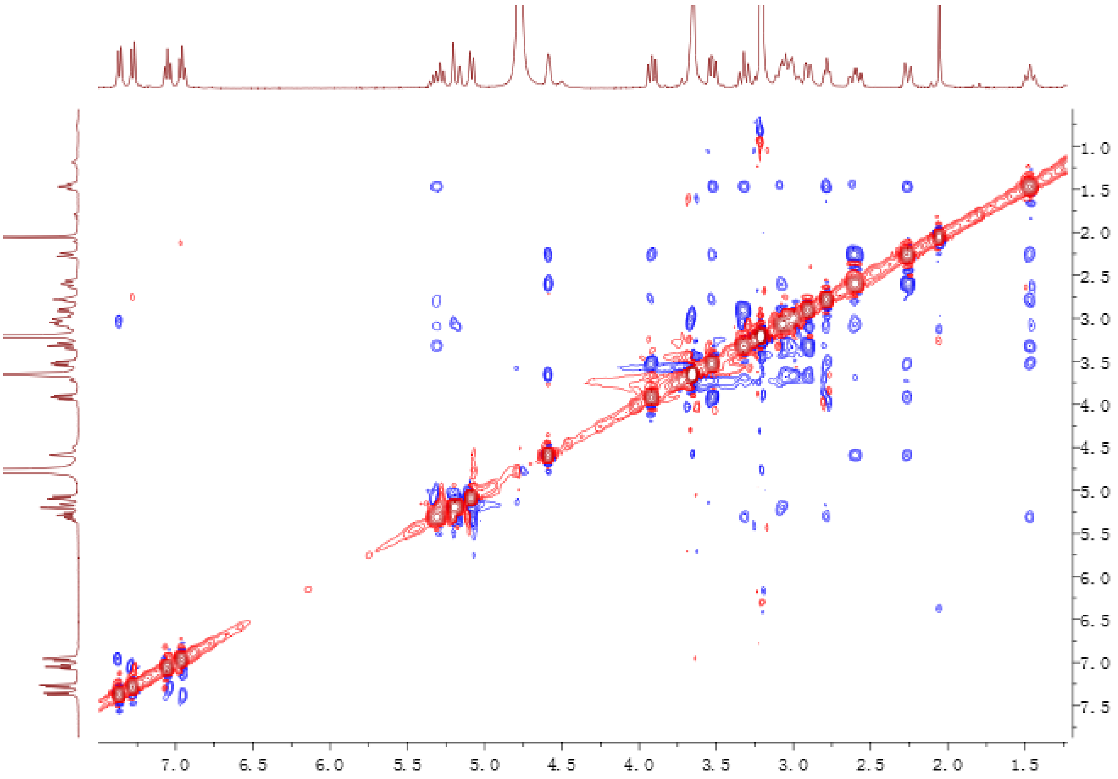

Fig. 24S ROESY spectrum of compound **3**(CD$_3$OD, 600MHz)

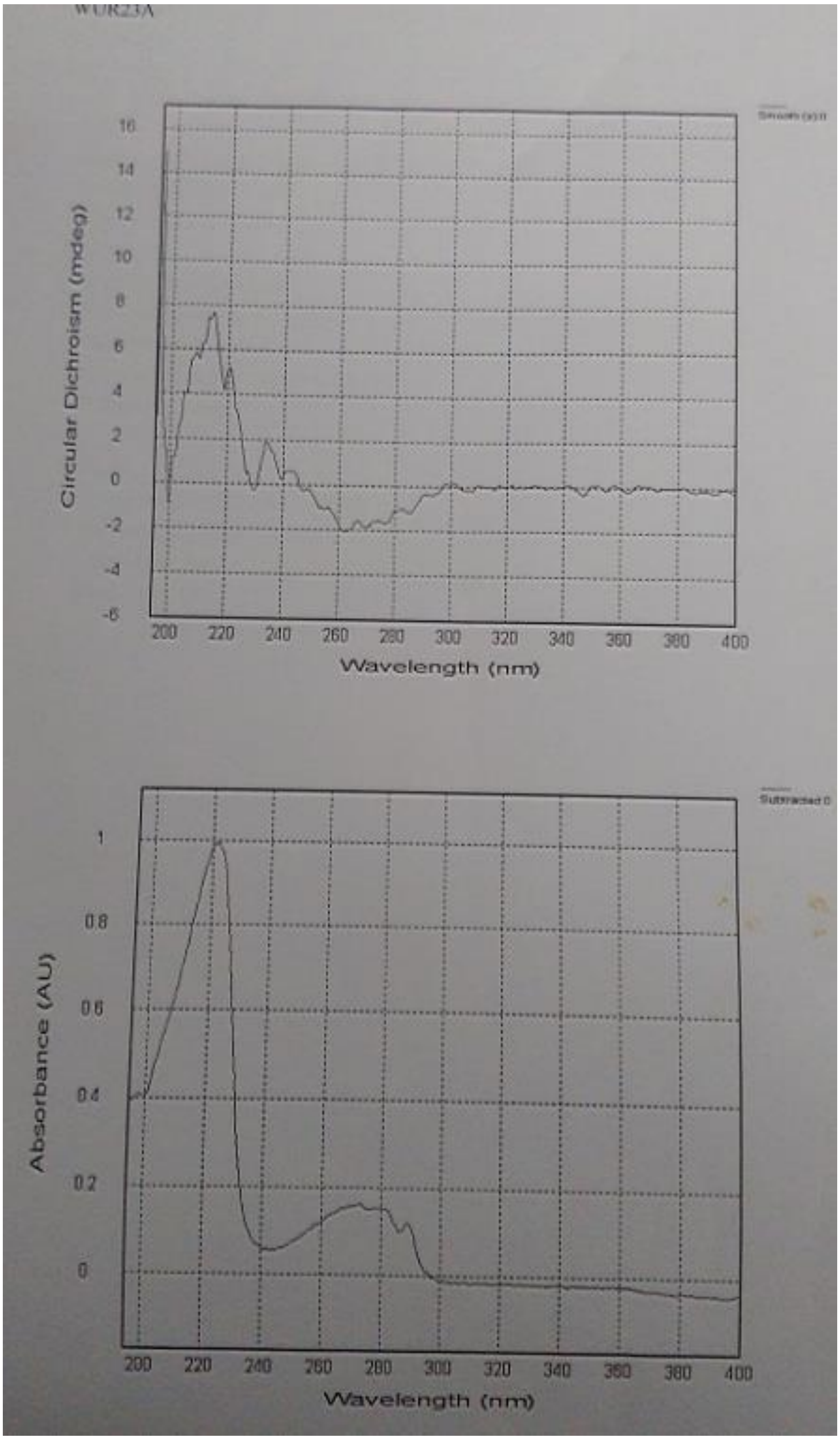

Fig. 25S ECD spectrum of compound **3**

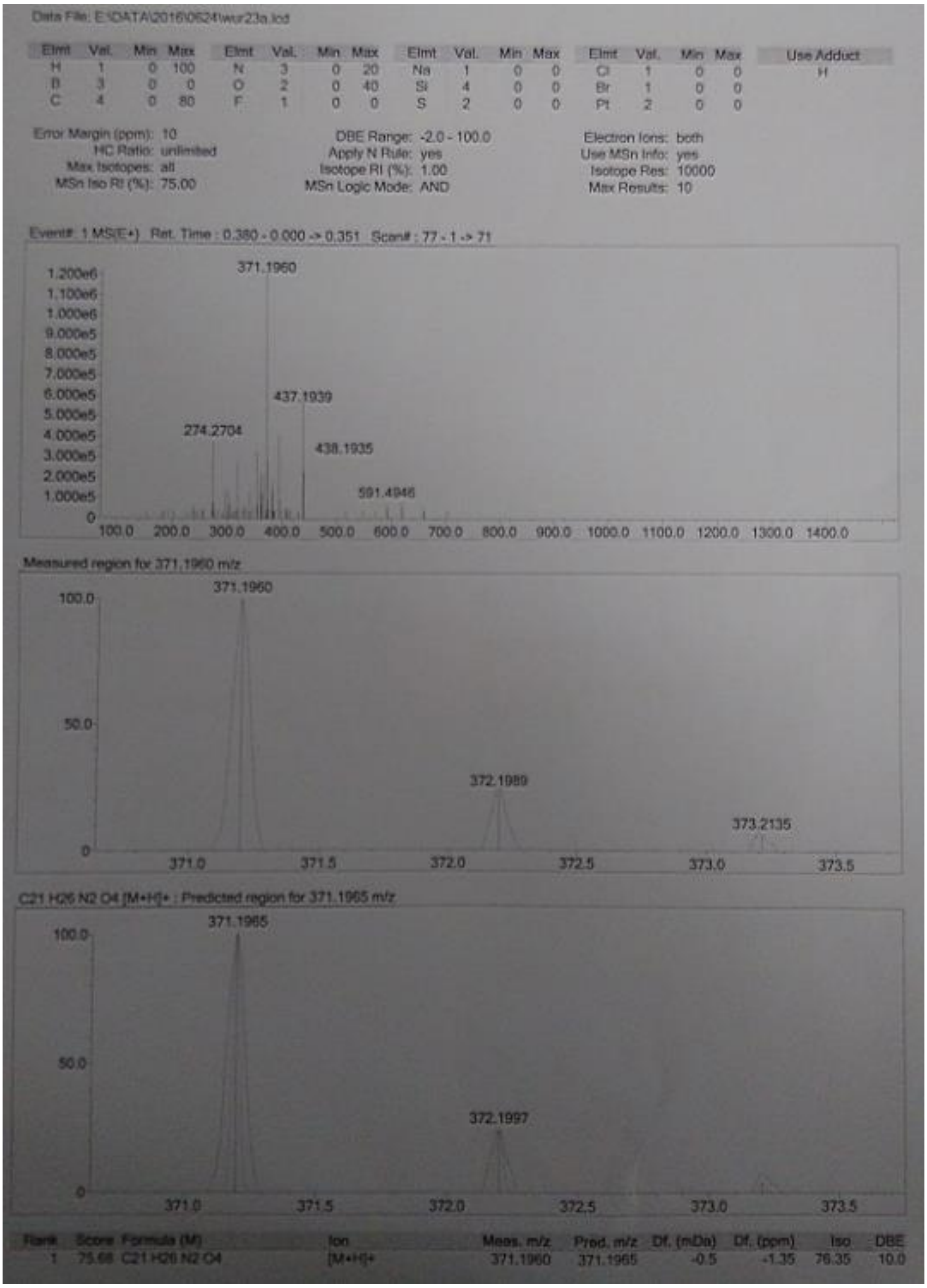

Fig. 26S HRESIMS spectrum of compound **3**

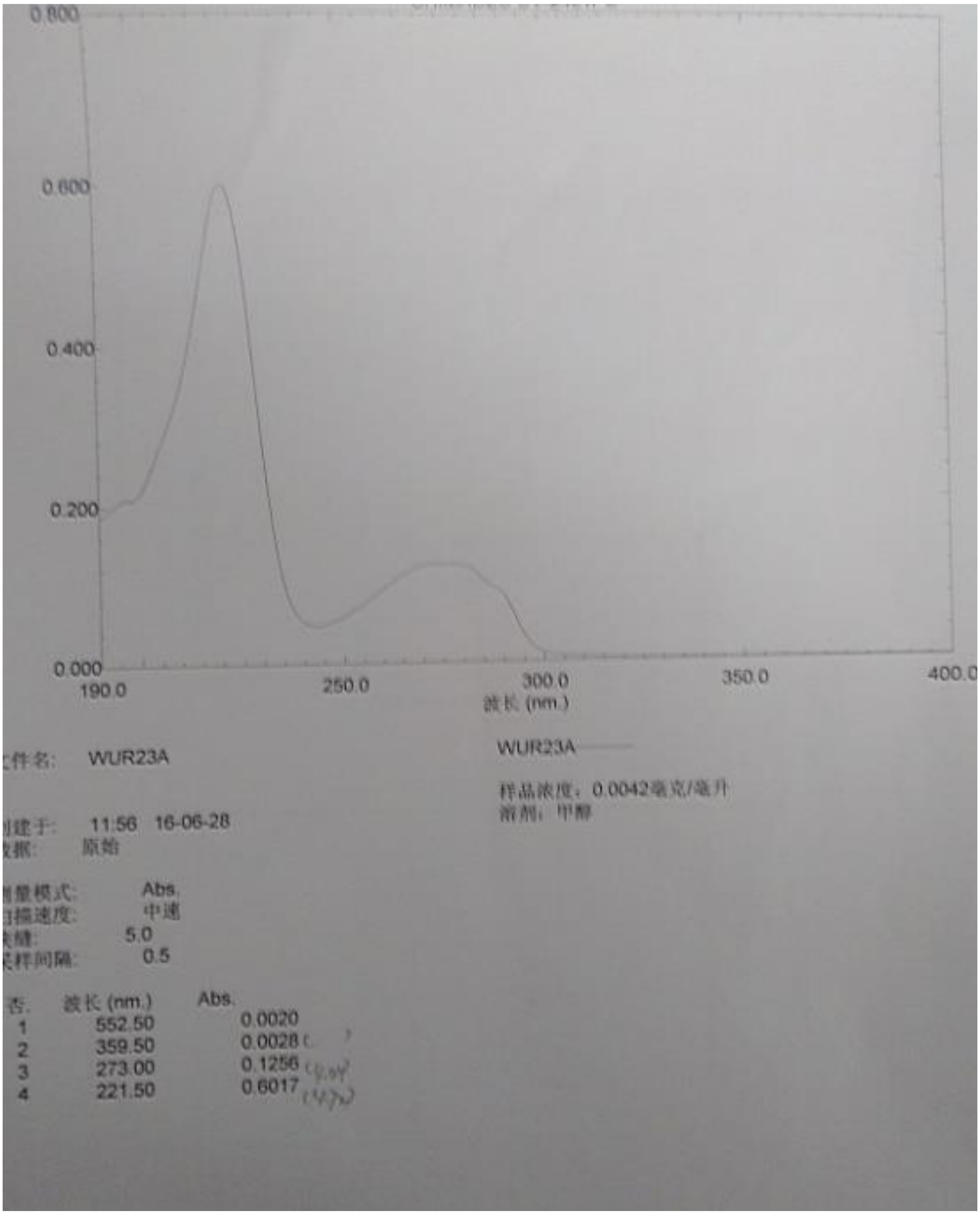

Fig. 27S UV spectrum of compound **3**
